# RNA-Based Therapeutics: From Antisense Oligonucleotides to miRNAs

**DOI:** 10.3390/cells9010137

**Published:** 2020-01-07

**Authors:** Sarah Bajan, Gyorgy Hutvagner

**Affiliations:** 1 Faculty of Science, University of Technology Sydney, Sydney, NSW 2000, Australia; 2 Health and Sport Science, University of Sunshine Coast, Sunshine Coast, QLD 4556, Australia; 3 School of Biomedical Engineering Faculty of Engineering and Information Technology, University of Technology Sydney, Sydney, NSW 2000, Australia

**Keywords:** miRNA, RNAi, drug delivery, Rna therapeutics, antisense RNA

## Abstract

The first therapeutic nucleic acid, a DNA oligonucleotide, was approved for clinical use in 1998. Twenty years later, in 2018, the first therapeutic RNA-based oligonucleotide was United States Food and Drug Administration (FDA) approved. This promises to be a rapidly expanding market, as many emerging biopharmaceutical companies are developing RNA interference (RNAi)-based, and RNA-based antisense oligonucleotide therapies. However, miRNA therapeutics are noticeably absent. miRNAs are regulatory RNAs that regulate gene expression. In disease states, the expression of many miRNAs is measurably altered. The potential of miRNAs as therapies and therapeutic targets has long been discussed and in the context of a wide variety of infections and diseases. Despite the great number of studies identifying miRNAs as potential therapeutic targets, only a handful of miRNA-targeting drugs (mimics or inhibitors) have entered clinical trials. In this review, we will discuss whether the investment in finding potential miRNA therapeutic targets has yielded feasible and practicable results, the benefits and obstacles of miRNAs as therapeutic targets, and the potential future of the field.

## 1. Introduction

### 1.1. Therapeutic Nucleic Acids

The majority of human diseases are influenced by genetic factors [1]. Therefore, one therapeutic avenue, gene therapy, substitutes the disease-associated gene with the ‘healthy’ version of the gene or gene products. The first approved human gene therapy treatment was approved in 1990 to treat adenosine deaminase deficiency [2]. The advantage of gene therapy is that it can successfully cure genetic diseases as demonstrated in 2002, with patients with severe combined immunodeficiency (SCID) [3]. Currently, gene therapies for more complex disorders like neurodegenerative Alzheimer’s disease [4] and polygenic cancers are in development [5].

DNA-based therapeutics, however, are not restricted to gene replacement [6]. DNA is also widely used for genetic immunization. DNA vaccines are currently restricted to veterinary clinics. However, clinical trials are ongoing for human use and hope to offer protection against malaria, HIV, influenza, tuberculosis and Ebola [7].

The first nucleic acid drug to get United States Food and Drug Administration (FDA) approval was fomivirsen (Vitravene), developed for immunocompromised patients suffering with cytomegalovirus (CMV) retinitis [8]. Fomivirsen is an antisense DNA oligonucleotide that is complementary to a region of the CMV transcript, preventing the replication of the virus [9]. Several DNA-based drugs have been approved since and this is still an active field of research. However, many biopharmaceutical companies are focusing on another nucleic acid for its therapeutic potential—RNA.

### 1.2. The Emergence of RNA-Based Drugs

Due to developing sequencing technologies and high-powered statistical and experimental approaches, accurate identification of specific disease-causing genetic variants and genes is becoming more achievable [10]. This has been a significant challenge of the genomics field, especially when determining the causal mechanisms of polygenic diseases, their risk variants and the diversity of cell types and environments [10].

Traditional small molecule drugs combat disease pathology by modulating the downstream pathways of a disease-causing gene. However, a more accurate and efficient treatment strategy that is currently emerging in the clinic is to modify the gene product at fault, by targeting the DNA, or the RNA precursors using genetic therapies.

Currently, many pharmaceutical and biotech companies are developing RNA-based therapeutics to specifically regulate disease-causing genes and their variants. A handful of RNA-based products have successfully been approved for use in the clinic, with many more in varying stages of the drug development pipeline. The majority of these products are synthetic, non-coding RNAs (ncRNAs) [11,12].

Endogenous ncRNAs are encoded in the genome and have essential functions in the regulation of gene expression, and due to the increasing accuracy and sensitivity of sequencing technologies, we have a rapidly expanding understanding of the specificity of their expression. Many ncRNAs are differentially expressed in disease states and are thought to contribute to disease development and progression. Therefore, they have great potential as diagnostic markers, therapeutic targets or drugs [13].

**Figure 1:**
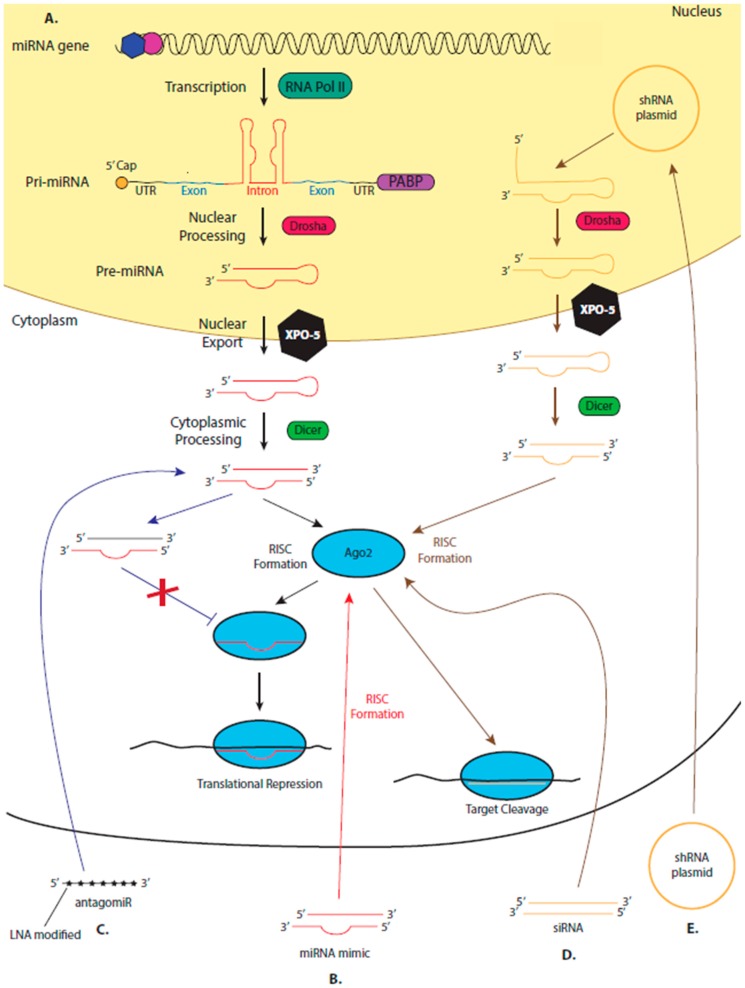
Mechanisms of RNA-based therapeutics that are dependent on the endogenous microRNA (miRNA) pathway. (**A**) miRNAs are encoded in the genome, often in the intron of protein-coding genes. The transcript, produced by RNA polymerase II, containing the miRNA forms a characteristic stem-loop structure which is processed in the nucleus by an RNases III enzyme, Drosha, to form an RNA hairpin (approx. 70 nucleotides) called the pre-miRNA. Pre-miRNA moves into the cytoplasm via exportin-5 (XPO-5), where it is further processed by Dicer, producing a double-stranded miRNA–miRNA* duplex. One strand of this duplex is loaded onto an Argonaute (Ago protein) to from the RNA-induced silencing complex (RISC). The other strand of the duplex (the passenger strand) is degraded. RISC is guided by the loaded miRNA strand which imperfectly binds to complementary sites commonly found in the 3’ untranslated region (UTR) of target mRNAs. RISC inhibits the translation of the bound mRNA and can cause deadenylation and degradation of the targeted transcript. Therapeutic miRNA mimics (**B**) are synthesized as miRNA duplexes. Upon entry into the cell, one strand binds to an endogenous Ago protein forming RISC, while the passenger strand degrades. The synthesized miRNA acts as a guide, directing the RISC to the therapeutic target, and inhibiting its translation. (**C**) AntagomiRs are single-stranded, synthesized, modified RNA molecules which are complementary to an endogenous miRNA. Upon entry into the cell, the antagomiR will bind to its target miRNA, preventing the miRNA from being loaded onto an Ago protein and forming RISC. (**D)** Once therapeutic siRNA duplexes enter the cell, one strand is loaded onto an Ago2 protein forming RISC. RISC is directed to the target mRNA by the loaded siRNA which binds with 100% complementarity to its target, Ago2 then cleaves the transcript. (**E**) DNA plasmids designed to encode short hairpin (sh) RNA enter the cell nucleus, where they are transcribed, producing an RNA with a characteristic stem-loop structure, allowing the RNA to enter the endogenous miRNA biogenesis pathway.

Despite being lauded as a potential drug target or therapeutic nearly since their discovery in 1993 [14,15], a microRNA (miRNA)-based drug is yet to reach the clinic. miRNAs, a class of ncRNA, are small (~21 nucleotides), single-stranded RNA molecules that primarily regulate post transcriptional stages of gene expression. The miRNA is loaded onto an Argonaute (Ago) protein to form the RNA Induced Silencing Complex (RISC) [16]. The RISC is the minimal effector complex for miRNA-mediated mechanisms of gene regulation. Guided by the miRNAs complementary binding to a site in a target transcript, commonly found in the 3’ untranslated region (UTR), RISC represses translation of the mRNA via one of several possible mechanisms (Figure 1) [17]. Around 2000 human miRNAs have been identified [18], each with a distinct expression pattern and a specific set of target miRNAs. Therefore, almost every biological process is controlled by miRNA-mediated mechanisms of regulation [17,19].

Due to their extensive influence on gene regulation, miRNA function and expression are spatially and developmentally controlled, which is essential for defining and maintaining cellular differentiation and identity. Therefore, it is not surprising that miRNA expression is altered in most disease states and has been found to play major roles in the development and progression of several diseases [20,21,22,23,24] and are actively being investigated and developed for clinical applications [25].

In this review, we will discuss the suitability and limitations of RNA-based drugs, and why miRNA therapeutics, thus far, have had limited success.

## 2. RNA Therapeutics: An Expanding Repertoire

The many functions of RNA, not only as a messenger of genetic information, but as essential and specific regulators of numerous steps of gene expression, are becoming increasingly apparent. Our understanding of ncRNA-based regulatory mechanisms created opportunities to develop potential therapeutics that harness the endogenous regulatory machinery and is designed accurately and specifically to regulate the target of choice by complementary base-pairing. Many ncRNA-based mechanisms of gene regulation depend on small, relatively stable, RNA molecules (e.g., miRNA). As these molecules can also be isolated from endosomes and microvesicles [26] they have great potential for therapeutic use or as biomarkers for disease. 

A number of RNA drugs have been developed to combat polygenic diseases like different kinds of cancer [27]. Additionally, many studies have focused on combatting viral infections with RNA therapies. Currently, most viral infections do not have specific drugs, and rely on pre-emptive vaccination to combat the pathogen. Therefore, RNA-based solutions are attractive, as there is the potential to directly and specifically halt expression of the viral genes required for the infection to spread [28].

### 2.1. Common RNA Drugs

The majority of RNA-based drugs used in the clinic or currently in development are antisense oligonucleotides (ASOs) or doubled-stranded RNA molecules called short interfering RNAs (siRNAs). ASOs and siRNAs both bind target mRNAs, or pre-mRNAs, via complementary Watson-Crick base pairing, but differ in composition and modes of action.

#### 2.1.1. Antisense Oligonucleotide

ASOs are single-stranded, highly-modified, synthetic RNA (or DNA) sequences, designed to selectively bind via complementary base-pairing to RNA which encodes the gene of interest, and have been tested in a number of disorders [29]. The binding of the ASO to the target can trigger a range of outcomes when bound to its complementary target [29]. These outcomes range from altering mRNA processing to degrading the target transcript. Synthesized ASOs can: bind to complementary sequences in pre-mRNA, altering the recruitment of splicing factors to the molecule, regulating splicing events; bind to mature mRNA and prevent its attachment to the ribosome, blocking protein translation or can recruit RNase H to the target transcript, which will then be degraded [30].

#### 2.1.2. Short Interfering RNA 

By contrast, short interfering RNA (siRNA), a small, double-stranded complex, which triggers the RNAi pathway. The RNAi pathway evolved as a natural cellular defence mechanism against RNA viruses, which identifies pathogenic double-stranded RNA molecules and targets them for cleavage [31]. One strand of the siRNA duplex is loaded onto an Argonaute 2 (Ago2) protein, forming RISC. Once RISC binds to the target mRNA at specific, perfectly complementary, binding sites, Ago2 cleaves the mRNA, leading to degradation of the transcript and, therefore, inhibiting its translation [32]. siRNA binding to its target is highly selective and can discriminate between sequences that differ by a single nucleotide [33]. This specificity in binding makes siRNA suitable therapeutic tools.

However, in humans, and other mammals, the RNAi pathway does not naturally occur. Instead, endogenously expressed human Ago proteins bind to small RNA molecules encoded in the human genome, called miRNAs, forming RISC, which has essential functions in gene regulation. However, as the RNAi and miRNA pathway share common proteins (such as Ago2) the proteins of the miRNA pathway can be used by synthetic siRNAs to bind to and cleave their targets (Figure 1). Introduced siRNA drugs compete with endogenous miRNAs for Ago2 binding, a limitation of all siRNA drugs. Therefore, the concentration of the RNA drug must be carefully optimised, as excess may saturate the RISC machinery, inhibiting its normal regulatory functions [34,35,36]. Despite this, the RNAi pathway can successfully be induced in humans and used to therapeutically to lower gene expression by adding synthetic siRNA to cleave its complementary target. Approximately twenty siRNA-based therapeutics have reached the clinical trial stages of drug development [37].

When using synthetic, therapeutic siRNA to inhibit the replication of an invading pathogen, there is a chance that the targeted pathogen becomes resistant to treatment or escape mutants form. Targeting more than one gene of the pathogen or using different siRNA duplexes that target the same transcript can help reduce this risk. However, this increases the possibility of off-target effects. Short hairpin RNAs (shRNAs), RNA molecules that are processed by endogenous machinery to form siRNA duplexes, that encode two or more siRNAs have shown promise in delivering a multiplexed approach to treatment (Figure 1) [38,39].

siRNA-based drugs are not limited to treating infections. Many in the drug development pipeline target disease-causing genes and gene variants involved in a wide variety of diseases including cancers, inflammatory disorders and neuropathies [37].

While many RNA-based drugs are in development, there are still significant barriers to efficient RNA-based treatment strategies including delivery of the drug to a particular site or tissue, off-target effects and longevity of the treatment.

## 3. Delivery of RNA Drugs

As regulation of gene expression is a highly dynamic process, dependent on tissue, environment and development, it is desirable to only regulate the intended drug target in disease-affected tissues. Targeting an RNA-based therapeutic to a specific tissue depends on the drug leaving the circulatory system, entering the appropriate cells, and, if contained in a carrier vessel, being released into the cytoplasm, before being filtered by the kidneys and excreted. While in circulation, the therapeutic is under threat from phagocytosis and destruction by immune cells. 

**Figure 2:**
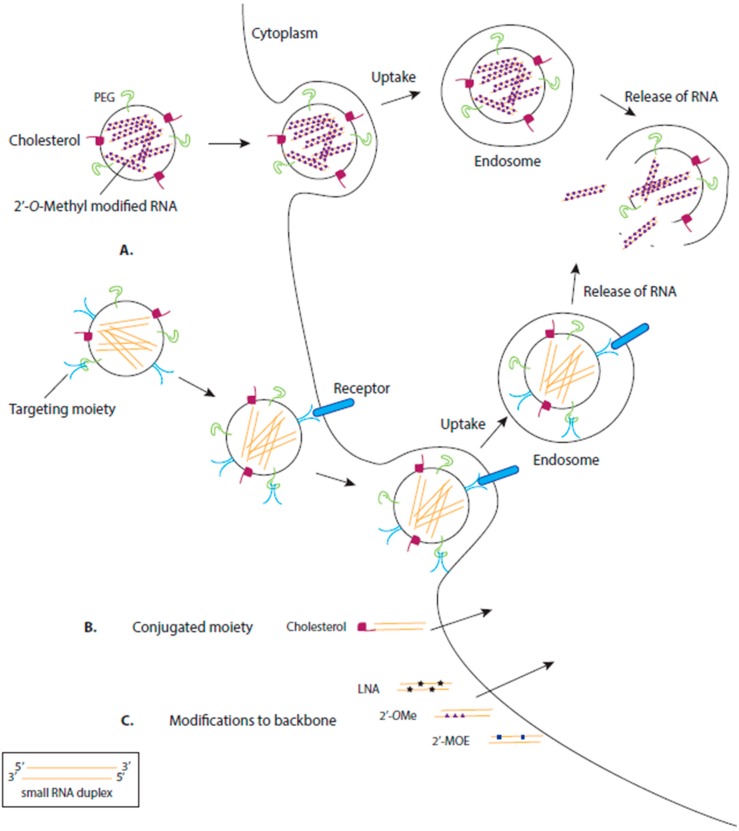
Common Delivery Methods for RNA-based Therapeutics. (**A**) RNA-based therapeutics are often encapsulated, or attached on the surface of, nanoparticles to aid delivery of the drug into the cell. These nanoparticles are often modified with moieties such as cholesterol or polyethylene glycol (PEG) which aid uptake of the nanoparticle via the cell membrane. Some nanoparticles are directed to particular cells by the addition of a targeting moiety, often a ligand for a cell surface receptor specifically expressed on the target cell. Commonly, the nanoparticle enters the cell via endocytosis, forming an endosome, which, after environmental changes (e.g., lowered pH), degrades, releasing the RNA therapeutic into the cell. (**B**) Alternatively, some RNA therapeutics are directly conjugated to moieties to aid their transport across the cell membrane, e.g., cholesterol (**C**) Synthesized RNA therapeutics can be chemically modified to increase their stability and binding affinity and decrease their toxicity. LNA: locked nucleic acid (2′4′-methylene; 2′*O*Me: 2′-*O*-methyl; 2′MOE: 2′-*O*-methoxyethyl.

RNA-based drugs are more easily introduced to cells as they lack the secondary or tertiary structures that large RNA molecules may form. However, these RNA-based drugs can be degraded by nucleases, have difficulty crossing the cell membrane as they are negatively charged and can trigger an immune response. To ensure efficient uptake of the drug in the desired location, while minimising toxicity, a variety of selective delivery agents are in development. Additionally, modifications to the chemical structure of the RNA help to overcome these hurdles (Figure 2) [40].

### 3.1. Chemical Modification of RNA

RNA molecules are inherently quite unstable, as they have a 2′ hydroxyl (OH) group [41]. Chemically modifying the base, sugar or backbone of a synthesized RNA molecule aids stability, increases their resistance to nucleases, improves efficiency and target specificity, or helps delivery into a cell [42,43]. Incorporation of 2′-*O*-methyl (2′OMe)-modifications in selective uridine or guanosine nucleosides into the passenger strand of an siRNA duplex eliminates cytokine induction, toxicity and off-target effects with can occur with unmodified siRNAs [44]. Over modifying RNA, however, can have toxic effects or render the molecule less efficient [45].

Synthetic RNA-based oligonucleotides often contain 2′OMe, 2′-fluoro, 2′-*O*-methoxyethyl (2′MOE) or 2′4′-methylene (locked nucleic acids (LNAs)) ribose sugars (Figure 2) [46,47,48]. These modified RNA-based oligos have an increased affinity to bind to and inhibit their target (e.g., target miRNA) in vivo, are more resistant to degradation and have increased bioavailability [49]. Furthermore, several animal studies have concluded that modified RNA-based oligonucleotides are specific, stable and non-toxic when delivered intravenously [50,51].

Some RNA-based drugs are modified with a 3′ cholesterol (Figure 2) [52]. Cholesterol is an essential component of animal cell membranes, is an efficient transmembrane transporter and is not toxic. The addition of a cholesterol moiety to siRNA will aid association of the siRNA to lipoprotein particles, lipoprotein receptors and transmembrane proteins. Different cholesterol conjugates can target the siRNA to specific tissues [53]. For instance, siRNA conjugated to a cholesterol that associates with high density lipoprotein (HDL) will preferentially target the liver, kidney and the intestines. Low density lipoprotein (LDL) associated cholesterol will target the liver and is dependent on the liver cells expressing LDL receptors. Lipid-conjugated siRNAs are efficiently delivered to cancer cells [54] and have been found to efficiently decrease HIV-1 replication in infected cells [55].

Systemic delivery of RNA-based therapeutics can be administered via intravenous injection or infusion. Delivery strategies in this case can be either active or passive [56,57]. Passive strategies take advantage of the function of organs like the liver, spleen and lymph nodes to internalise accumulated particles, causing the RNA-based drug, and any associated carrier particle to concentrate in these organs [56,57]. The blood-brain barrier poses a significant hurdle to RNA-based drug delivery to the central nervous system (CNS). RNA therapeutics designed to treat neurological diseases such as nusinersen approved for use to treat spinal muscular atrophy require intrathecal delivery [58]. However, due to the lack of specific targeting of the drug, there is an increased chance of off-target effects with this delivery method. Injected delivery direct to the site of the pathology (e.g., a malignant tumour) can enhance target specificity and efficacy. However, many tissue- and cell-specific delivery methods are in development [59].

### 3.2. Delivery by Ligand-Based Targeting Molecules

Some RNA-based drugs in development are ‘self-delivering,’ which means that the therapeutic siRNA duplex requires no additional delivery vehicles for targeted distribution in the body. A self-delivering siRNA designed to treat malignant melanoma, conjugated to a 3′ cholesterol moiety via a tetraethylenglycol (TEG) linker, targets T cells [60].

However, many RNA-based drugs require specific nanoparticle delivery systems (Figure 2). For cell-specific delivery of RNA-based therapeutics, one strategy being investigated is attaching an aptamer to the drug. An aptamer is a nucleic acid sequence with a unique 3D structure that has a high affinity to a chosen cell target. Aptamer-siRNA molecules have been generated as one covalently bound polymer which aids receptor-targeting and siRNA delivery. This delivery method was tested for targeted delivery using an aptamer designed with high affinity for prostate-specific membrane antigen (PSMA) expressed on the surface of prostate cancer cells. The aptamer-siRNA molecule was internalised into the cell, allowing the RNAi directed silencing of the siRNA target [61]. Furthermore, in vivo tests revealed that the addition of a 20 kDa polyethylene glycol (PEG) moiety to the 5′ end to strand of an siRNA duplex increased circulation and bioavailability of the aptamer-siRNA molecule [62]. Targeting aptamers have also been designed to target HIV-1 specific proteins to selectively deliver siRNAs into HIV-infected cells, allowing for a more potent inhibition of HIV target transcripts [63].

Antibodies are also used to direct siRNAs to specific cells, by binding specific surface receptors to ensure directed delivery of the therapeutic. These antibodies were modified to allow for a charge interaction with siRNA at their C-terminus, forming an antibody-siRNA delivery complex that specifically bound to the T cell surface protein CD7 to suppress HIV infection [64]. To enhance the targeting antibody, cell-penetrating peptides, less than 30 aa, can also be linked to aid movement of drug into specific tissues [65].

As discussed (Section 3.1), another promising ligand for siRNA delivery is cholesterol. As cholesterol is non-polar, it easily incorporates into cell membranes and other lipid-bound vesicles. The addition of cholesterol to siRNA increases stability, cellular uptake and membrane fusion [52].

### 3.3. Delivery by Lipids

Encapsulating the RNA-based drug in a lipid vesicle is another delivery strategy that protects the drug from degradation and facilitates its transfer across the cell membrane (Figure 2). However, the endogenous endosomal pathway of the cell often causes internalised vesicles to be degraded. To overcome this challenge, synthetic cationic lipids have been designed to allow for the release of the contained drug (i.e., siRNA) once internalised into a cell. These synthetic lipids are neutralised by the cellular anionic lipids, releasing the siRNA and allowing efficient target silencing. 

Further modifications to the liposome can enhance drug delivery specificity and efficiency. Surface-attached antibodies have been demonstrated to increase the specificity of liposomal carriers of siRNAs, directing these nanoparticles to specific cells, such as leukocytes [66]. The addition of PEG-lipid conjugates to the liposome forming stable nucleic acid lipid particles (SNALPs) enhances plasma concentration, cell internalisation and the release of the drug [67,68,69].

### 3.4. Delivery by Polymers

Synthesized polymers are an alternate strategy for forming RNA-based drug nanoparticles. The polymers must be biocompatible to prevent cytotoxicity and accumulation of the polymer in cells and tissues. Polymer delivery systems envelope the RNA-based drug, providing protection for degradation, and provide target specificity via the addition of surface ligands [70]. However, the delivery efficiency of these carriers is low. As with other delivery methods, modifying the polymer with PEG enhances stability, specificity and solubility of the polymer [71]. Similar to liposome delivery, this system requires a mechanism of releasing the drug from the polymer once the nanoparticle has been internalised. Some polymers rely on the reductive environment of the cytoplasm to cleave disulphide linkages in the polymer, releasing the bound drug [72]. 

### 3.5. Delivery by Viral Vectors

Viral vectors have long been used to deliver and, in the case of retroviruses and lentiviruses, incorporate transgenes into the genome of host cells. Viral vectors can infect a wide variety of cell types, with lentiviruses also being able to infect senescent cells [73]. Viral vectors have been successfully modified for efficient packing and expression of the transgene and is also self-inactivating [74,75,76]. Delivery of RNA-based drugs by viral vectors occurs via the packing of short hairpin RNA (shRNA) expressing plasmids into a lentiviral vector. The shRNA sequence is integrated into the host genome and shRNA expression is under promoter control for long-term expression. The expressed shRNA is processed by endogenous proteins that generate miRNAs to form siRNA, which then targets transcripts by forming RISC.

Strategies which include genome integration carry the risk of off-target effects, which can occur if integration occurs in a coding or regulatory sequence within the genome. One way to reduce the risks of this is via an artificial tether, which directs the vector to a safe and target-specific genomic site for integration [77].

Transient expression of shRNA via viral delivery has been achieved with adenoviral and recombinant adeno-associated viral (rAAV) vectors. rAAV vectors have the advantage of being non-pathogenic. Therefore, they cannot replicate in host cells, allowing for an increased control over the treatment delivery and longevity. This delivery strategy has been used to down regulate genes in various cancer cells [78].

### 3.6. Delivery by Bacterial Mini Cells

Bacterial mini cells are spherical cells that lack chromosomal DNA and, therefore, are unable to proliferate. Recombinant mini cells can be used as a targeted drug delivery system [79]. The mini cells, containing miRNA, siRNA, or plasmid encoding shRNAs are directed to specific cells or tissues via surface expressing antibodies. 

While this delivery method did not cause toxicity in animal studies [80,81], its bacterial origin may trigger an inflammatory response in patients, mediated by the Toll-like receptors, if systemically administered [82]. Many RNA modifications and delivery strategies discussed here aim to reduce the immune response, which is triggered by unmodified RNA molecules [83]. However, there may be some benefit to triggering a localised immune response. siRNA function can be enhanced by interferon (IFN) induction, which the immune system can harness to boost apoptosis of metastatic tumour cells [84]. Introducing a miRNA-like non-pairing bulge in the passenger strand of an siRNA complex increased immunostimulatory activity of human immune cells, without compromising the silencing efficiency of the siRNA. The associated increased cytokine production, therefore, has a potential application in antiviral therapeutics [85].

## 4. RNA-Based Therapeutic Success

### 4.1. Modifying Alternative Splicing to Increase Protein Production

Spinal muscular atrophy (SMA) is an autosomal recessive, degenerative, neuromuscular disorder that causes the loss of spinal motor neurons leading to muscle wasting [58]. SMA is one of the most common causes of infant mortality, with a carrier rate of 1:50 and in incidence of 1 in 10,000 births [86]. SMA develops due to low levels of survival motor neuron (SMN) protein, which is caused by deletions or inactivating mutations within the *SMN1* gene. *SMN2* is an almost identical gene to *SMN1*, which differs by a single nucleotide at the beginning of exon 7 [87]. This variation weakens a splice site in SMN2. Therefore, most of the time (85%–90%), *SMN2* gene produces a transcript which lacks exon 7. When the shortened transcript is translated, SMN2 is expressed as a truncated, unstable protein (SMN2∆7) [87,88]. The higher the amount of functioning, full length SMN protein produced, the less severe the disease [89,90]. Therefore, genetic therapy focused on boosting SMN2 splicing to express a full length SMN protein.

Nusinersen (Spinraza^TM^, Biogen) is the only approved treatment for SMA in the USA and Europe (2017) [91], due to patients experiencing improved motor function, a slowing of disease progression and few side effects. The progression of nusinersen into clinical use was well received by the field, as demonstrated by a standing ovation during the 2017 RNA conference, following the announcement it recieved FDA approval.

**Figure 3:**
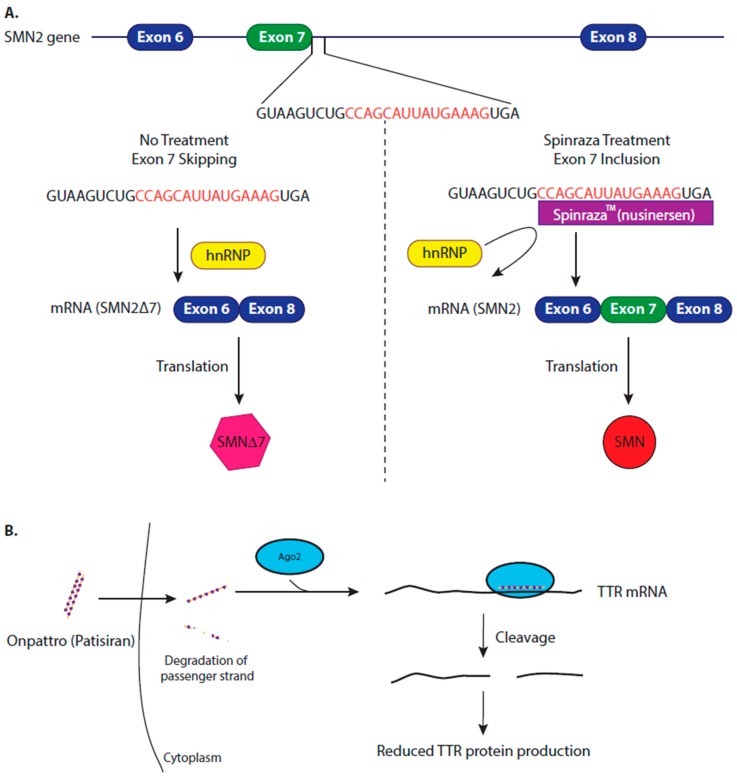
Mechanism of approved therapeutics. (**A**) Nusinersen regulates splicing of the Survival Motor Neuron (*SMN)* 2 gene to treat patients with spinal muscular atrophy (SMA). Due to weak splice site, masked by the binding of hnRNP, the *SMN2* gene usually produces a truncated transcript lacking exon 7, which, when translated, produces a non-functional and unstable protein (SMN2∆7). Nusinersen (Spinraza^TM^, Biogen) is an antisense oligonucleotide (ASO) therapy that binds, via complementarity, to SMN2 pre-mRNA, displacing hnRNP, exposing the splice site and increasing the inclusion of exon 7, forming a full-length, mature SMN2 transcript. Once translated, this produces a full-length, functional SMN protein, which improves patient’s motor neuron function and slows disease progression. (**B**) Patisiran (Onpattro) reduces the production of transthyrethin (TTR) protein to reduce the formation of amyloid fibrils in hereditary transthyretin-mediated (hATTR) amyloidosis. Mutations in the *TTR* gene causes misfolding of the TTR protein, the misfolded protein aggregates into amyloid fibrils. Patisiran is a synthesized siRNA therapy, which is 100% complementary to a specific sequence in the 3′ UTR of the TTR mRNA. Once Patisiran enters the cell, one strand of the short interfering RNA (siRNA) duplex is loaded onto an Ago2 protein, forming RISC. RISC binds to the TTR transcript, which is subsequently cleaved by Ago2, therefore reducing TTR protein production, preventing further amyloidosis and improving patient’s quality of life.

Nusinersen is an ASO that binds to a regulatory sequence in intron 7 of the SMN2 pre-mRNA molecule, a site that is normally occupied by the heterogeneous nuclear riboprotein (hnRNP A1/2), masking the regulatory sequences required for exon 7 splicing. The binding of nusinersin to this site, displaces the hnRNP A1/2 complex, promoting the inclusion of exon 7 in the mature SMN2 mature mRNA sequence, consequently increasing the levels functional SMN protein (Figure 3A) [92,93].

Similarly, an ASO is also approved for use in Duchenne Muscular Dystrophy (DMD). DMD is a rare, X-linked recessive disorder characterised by a progressive loss of muscle tissue [94,95], caused by deletions within the dystrophin gene. Deletions in this gene generates a premature stop codon, creating a truncated product which is degraded by nonsense mediated decay. Therefore, no functional dystrophin protein is produced in these cells. ASO therapy has focused on exon 51 in the dystrophin gene, redirecting the splicing machinery away from the exon, in order to restore the open reading frame of the mature mRNA transcript. This regulation of alternative splicing will generate a milder phenotype of the disease, although this is only amenable to 13% of these patients. Drisapersen, a 2′-*O*-methyl phosphorothioate oligonucleotide, was the first exon-skipping ASO therapy for DMD in clinical development [96,97]. However, patient improvement in phase 3 clinical trials was not sufficient for regulatory approval [98]. Exondys 51^TM^ (Eteplirsen), a morpholino ASO that selectively binds to the exon-intron splice site at the beginning of exon 51, restoring the open reading frame, which results in the production of a truncated, but functional dystrophin protein. Due to limited clinical outcomes, Eteplirsen controversially received approval from the FDA for clinical use in 2016 [99].

Several other ASO drugs are currently in clinical trials underpinning the great promise of future successful treatments using this tool, e.g., for Huntington’s disease (NCT02519036), amyotrophic lateral sclerosis (ALS) (NCT02623699) and acute non-arteritic anterior ischaemic optic neuropathy (NCT02341560).

### 4.2. Inhibiting Protein Production to Reduce Amyloidosis

In an exciting breakthrough for RNA-based therapeutics, the first short interfering RNA drug, Onpattro (patisiran) (Alnylam Pharmaceuticals), was granted approval for clinical use by the US FDA and within the EU in 2018 [100,101]. Onpattro is a new treatment option for patients with hereditary transthyretin-mediated (hATTR) amyloidosis [102,103], a rare, progressive, neurodegenerative and life threatening disease, with a median survival time of 4.7 years following diagnosis [104]. hATTR amyloidosis is caused by mutations in the transthyrethin (*TTR)* gene, which causes the TTR protein to misfold. The misfolded protein aggregates into amyloid fibrils which accumulates in multiple organs [105], causing heterogeneous clinical presentations which include polyneuropathy and cardiomyopathy [106,107]. TTR is mainly expressed in the liver and transports thyroxine and retinol-binding protein [108]. Liver transplantation is the standard of care for hATTR amyloidosis. However, this treatment is limited to availability of donors and comes with risks of immunosuppression in the patients [109].

Other treatment strategies which stabilise the TTR protein and prevent amyloid formation, have also been studied in hATTR amyloidosis patients. Difusinal, a nonsteroidal anti-inflammatory drug, increased survival time. However, patients suffered with serious side effects and, therefore, difusinal was not approved for clinical use. Tafamidis, a chaperone that stabilises the correctly folded TTR protein, delays degeneration of the neurons in hATTR amyloidosis and patients had an improved quality of life. Tafamidis is approved in Europe for treatment of hATTR amyloidosis.

Due to the limited success of stabilising the TTR protein, an alternative therapeutic strategy is to inhibit gene expression of TTR. Over 120 genetic variants of the TTR gene are associated with hATTR amyloidosis [110], which makes an siRNA a viable option for drug development as it can be designed to target all TTR transcripts, regardless of any specific mutation [111]. Onpattro is a lipid nanoparticle formulation of siRNA, delivered by intravenous injection, which is targeted to the hepatocytes. The siRNA binds the TTR transcript at a conserved site in the 3′ UTR of the TTR transcript, this triggers the cleavage, and subsequent degradation of the WT and mutated TTR transcripts (Figure 3B). After Onpattro treatment, synthesis of TTR protein is reduced in a dose-proportional manner (as measured by serum TTR levels), preventing further amyloidosis and promoting the clearance of the fibrils already present in the cells [112]. Clinical trials concluded that Onpattro resulted in significant improvement in patient’s quality of life and in clinical outcomes with 56% of patients showing an improvement after 18 months of treatment, in comparison to 4% of patients showing improvement with placebo treatment [111,113,114]. Onpattro also showed a consistent safety profile [103].

Another oligonucleotide-based drug for hTTR amyloidosis was also approved for clinical use in October 2018 in the EU and the US [115,116] as patients experienced delayed neuropathic disease progression after treatment. Tegsedi (inotersen) (Ionis Pharmaceuticals/Akcea Therapeutics) is a chemically modified 20 mer antisense oligonucleotide (ASO), which is complementary to a conserved region of the 3’UTR of the TTR transcripts present in both the wild type and disease-causing variants. Tegsedi’s mode of action is distinct from Onpattro, as chemically synthesized ASOs bind in a 1:1 stoichiometry to a complementary section of mRNA, causing degradation of the bound transcript by RNAse H [117]. RNAi, however, is catalysed by the Ago2 protein, and, therefore, one siRNA can cleave a large number of target mRNAs [118]. However, in comparison to siRNAs, ASOs enter the target cell of interest with a higher efficiency.

While some siRNA drugs are successfully advancing through the drug development pipeline, others have been withdrawn from further development. An example of this is Bevasiranib (OPKO Health), a 5-methoxy (CH_3_0) modified siRNA duplex designed to target vascular endothelial growth factor (VEGF), to treat diabetic macular edema or age-related macular degeneration (AMD) [119,120]. Bevasiranib reached phase 3 clinical trials. However, this study was halted due to poor performance. Studies with altered drug regimens and combination therapies are reportedly in the preparation stages.

Similarly, AGN211745 (Allergan and Sirna Therapeutics) entered clinical trials targeting VEGF receptor I (VEGFRI) to treat AMD [121]. However, this study was stopped before completion, despite acceptable safety reports of the drug, as patient improvement was less than expected.

## 5. miRNA Therapeutics

miRNAs are endogenous to the human genome and have an inherent and critical role in development and cell differentiation, proliferation, and survival [14,122,123]. miRNA mechanisms are essential in cell maintenance, homeostasis and responding to dynamic environments. Misregulation of miRNA expression and function due to genomic alterations or changes to miRNA biogenesis, such as mutations, deletions, amplifications, transcriptional changes and enzymatic differences, are important factors in disease development and progression [124,125,126,127]. Mature, single-stranded miRNAs bind to an Ago protein to form RISC, the same complex that is exploited by synthetic siRNAs to regulate gene expression. The miRNA-Ago complex binds to complementary sites in target transcripts and inhibits post-transcriptional stages of gene expression [17].

siRNAs perfectly bind to their targets with 100% complementarity and mediate cleavage of the transcript [128]. However, miRNAs imperfectly bind to mRNA, with a minimum binding requirement of nucleotides 2–8 of the miRNA, known as the seed sequence, to identify their targets [19]. Once bound, the miRNA regulatory complex represses translation of the target [124]. Due to the nature of their binding, miRNAs can bind to and inhibit a multitude of mRNA targets, creating a vast and intricate regulatory network. This unique ability of miRNAs, to target multiple mRNAs, potentially makes miRNAs very suitable therapeutic candidates [12,125,129].

Furthermore, as changes in miRNA expression can be indicative of mechanisms of disease development, progression and tissue of disease origin, quantifying miRNA changes in a patient could provide crucial insight required for diagnosing and tailoring treatment regimens to the individual [130,131]. As discussed earlier (Section 1.2), miRNAs are ubiquitously expressed, can be extracted from body fluids and quantification techniques are fast and robust. These attributes of miRNAs further increase their potential as essential therapeutic tools as we move towards precision medicine [132,133].

The advances in RNA chemical modifications and delivery systems have led to successful ASO and siRNA therapeutics and have also been applied to the development of miRNA drugs [129,134]. Despite several miRNA studies showing great therapeutic promise, some obstacles remain in this field, as a miRNA therapeutic has yet to reach the clinic. While therapeutics are still to emerge from the pipeline, the use of miRNA(s) as diagnostic tools has been more successful.

### 5.1. The Suitability of miRNAs as Diagnostic Biomarkers

The ideal biomarker is minimally invasive or non-invasive to obtain, easy to detect, stable, specific, robust and reproducible [135]. Clinical biomarkers promise not only to indicate a pathology is present, but also indicate the genetic origin of the disease, the stage of its development and the treatment which will have the best outcome. miRNAs are, therefore, ideal biomarker candidates, as they are ubiquitously expressed, can easily be isolated and sensitively and specifically quantified from tissue biopsies and body fluids by next generation sequencing (NGS), reverse transcription (RT) quantitative PCR or microarray [136,137,138,139]. Furthermore, miRNAs are stable, display cell-type specificity [140,141,142,143], and have physiological relevance, as distinct miRNA profiles have been identified in patient subgroups. There are, however, some known limitations of miRNAs as diagnostic biomarkers including suboptimal RNA extraction [144], detection assay variability [145,146,147] and non-standardised statistical analyses from miRNA clinical testing [148]. Additionally, due to genetic variability, miRNA expression and function differs in populations of people with different ethnic backgrounds, which not only may contribute to observed health disparities between populations but is also an essential consideration when assessing the suitability of miRNAs as diagnostic biomarkers [149,150].

Despite these limitations, identifying miRNA(s) as diagnostic biomarkers is a large and active field, in both the academic and industrial sectors. A small, but growing number, of these studies have successfully been translated into clinically available products, many of which are used to diagnose and/or prognose different cancer types and age-related diseases [25]. Most of these diagnostic tests investigate the expression levels of several miRNAs using libraries or panels, ranging from 10–19 miRNAs. Due to the polygenic and progressive nature of these diseases, it is not surprising that a suite of miRNAs is more suitable for accurate diagnosis. However, there are miRNA diagnostics currently in development which investigate the expression of a single miRNA, such as one for liver disease (miR-122, Quanterix) [151]. With many more miRNA-based diagnostic tests in development for a wide range of diseases, and with the increasing push towards precision medicine, it seems likely that more miRNA diagnostic panels will be available in the clinic in the near future.

The high sensitivity of miRNA measurement tools used in diagnostic panels means that in theory, the pathology can be accurately identified and, therefore, treated at an earlier stage than traditional diagnostic methods, increasing favourable patient outcomes. The majority of miRNA-based diagnostic tests consist of miRNA panels or libraries. The need to measure a suite of miRNAs to diagnose one condition underpins the complexity of miRNA involvement in disease development. However, as disease understanding increases, this information may help to tailor treatment regimens to the individual. 

### 5.2. The Suitability of miRNAs as Therapeutics

As the vast majority of human diseases involve the deregulation of multiple genes [152,153], modern pharmacology aims to regulate several targets in a multi-pronged approach to combatting disease. This also can help to solve the problem of redundant cellular pathways which can limit the efficiency of drugs that are capable of only regulating one target. Due to their unique expression patterns, their ability to target numerous transcripts [25], often in the same biological process, miRNAs can potentially regulate the expression of many genes in a tissue- or cell-specific manner [154]. miRNAs, therefore, have the potential to regulate an entire signalling pathway known to be misregulated in a pathogenic state, increasing their desirability in clinical applications. Conversely, the ubiquitous nature of miRNA binding, to perhaps as yet undiscovered targets, may have unanticipated and adverse off-target effects [45,155,156]. While RNA drugs are designed to exclusively bind to their intended target(s), the drug could bind to and regulate another gene. This chance may be heightened when designing miRNA-based drugs as the region of complementarity, which specifies its target, is limited to the eight-nucleotide-long seed sequence. The limited length of this sequence means it is unlikely to be uniquely present in the intended target, which may increase the chance of off-target effects. Furthermore, miRNA:mRNA interactions are incompletely understood as a well characterised miRNA:mRNA association displays poor seed region binding [157], while high seed sequence complementarity does not guarantee physiologically relevant regulation of the mRNA target [158]. Therefore, there is currently a relatively small number of experimentally validated miRNA:mRNA interactions and a need for more accurate bioinformatic prediction methodologies [159].

Many bioinformatic tools currently exist to make these predictions. However, as miRNA regulatory networks are highly complex, the accuracy rate of these predictions is low (approx. 26%) [160]. It is, therefore, critical to validate the predicted miRNA targets in vitro and in vivo, to assess their suitability as a drug target or therapeutic. Many high-throughput techniques have been developed to facilitate in vitro validation. However, the widely used in vivo animal models have significant limitations such as accurate disease replication and competing with endogenous miRNA(s). Identifying and validating the suitability of a miRNA as a therapeutic or target are still, therefore, major challenges in the field.

### 5.3. miRNA Mimics and AntagomiRs

As individual miRNAs can be either over or under expressed in different disease states, miRNA therapeutics aim to either boost a particular miRNA expression or reduce the level of a specific miRNA. 

In order to increase the level of a miRNA whose level has been lowered by the development or progression of disease [161,162], miRNA mimics are used. miRNA mimics are synthetically produced RNA molecules that have the same sequence as the under expressed endogenous miRNA. These mimics can re-establish normal expression and function of the miRNA of interest.

Short hairpin RNAs (shRNAs) can also be used as a miRNA replacement tool. These synthetic RNA molecules are processed by the endogenous processing machinery of the miRNA pathway into mature miRNAs [142,163,164]. As they are processed at a rate acceptable to the cell, they minimise toxicity, while still effectively regulating their targets [165,166]. Several miRNAs can be poly-cistronically encoded to be under the control of a single promoter, producing different functional regulatory complexes with low cytotoxicity [166,167,168], targeting a wide range of targets in one treatment.

In cancer, the majority of miRNAs are expressed at a lower level when compared to healthy tissue. Therefore, there is great investment in developing miRNAs as anti-tumour therapeutics. Many miRNAs regulate the cell cycle and are considered to have tumour-suppressing functions. Replacement of these miRNAs may complement or augment current cancer treatments. For example, normal miRNA regulation is critical to controlling the expression of oncogenes, e.g., let-7 regulates Ras, Myc and HMGA-2 [169,170]. p53 expression is promoted by the miR-34 family, which is commonly deleted from cancers. miR-15 and 16 are frequently deleted in B-cell lymphocytic leukaemia and prostate cancer. While other miRNAs are encoded at fragile sites in the genome where chromosomes can break, contributing to cancer development [164]. Furthermore, when genomic instability is a component of disease development, mutations accumulate in the genome. If a mutation occurred genomic sequences encoding miRNAs or their associated regulatory sites, this can have a drastic effect on the function of the miRNA, either eliminating its ability to bind to its targets, redirecting the miRNA to bind to and repress a new set of transcripts, or losing its tissue-specific expression pattern.

miRNA replacement therapy must deliver the drug at effective, but safe levels. Over expression of the drug can cause hepatotoxicity, organ failure and death [171]. As the drug depends on endogenous machinery to facilitate the action, and processing if applicable, of the drug, this limits the amount of the drug a cell can tolerate [165,171,172,173,174,175]. 

The potential toxicity caused by miRNA replacement therapies may be mitigated by using local delivery methods. A recent study ectopically re-expressed miR-215-5p in malignant pleural mesothelioma (MPM) cells which was delivered in vivo to tumour xenografts in a pre-clinical mice orthotopic MPM model. This increase in miR-215-5p activated p53 function, which induced apoptosis in a localised site [176].

Conversely, some specific miRNAs, termed onco-miRs, can increase tumour growth by inhibiting tumour-suppressing mechanisms [177]. The aim of drugs targeting these miRNAs would, therefore, be to repress their regulatory function.

AntagomiRs are used to suppress the function of a specific miRNA that is overexpressed and has a function in disease development/progression [161,162]. These are synthetic RNA molecules that will bind to and sequester the endogenous miRNA, preventing it from binding to and regulating the expression of its mRNA target.

miRNA function can also be repressed by introducing competitive binding sites for the miRNA. These binding sites can be designed into short, single-stranded, synthetic ASOs [29]. These ASOs contain chemical modifications to the backbone and sugar rings of the nucleotides increasing their resistance to nuclease degradation, its binding to plasma proteins to maintain stable serum concentrations, its complementarity to target RNAs and its ability to activate the innate immune system. The ASOs can be designed to contain high-affinity miRNA binding sites, to which the endogenous miRNA will preferably bind, and, therefore, sequesters the miRNA machinery away from the physiologically relevant targets. Furthermore, the ASO is specifically designed to prevent high turnover by the miRNA machinery. Such miRNA target decoys or sponges, could provide a more cost effective method of reducing activity of a specific, or set, of targeted miRNAs [178,179].

### 5.4. Leading the miRNA Therapeutic Field

No miRNA-based therapies have broken through the translation barrier and been FDA approved as yet. However, several potential therapies have reached phase I and phase II clinical trials (Table 1), with many more in clinical development. Many biotech companies are exclusively working on miRNA-based drugs such as Miragen, Synlogic and Regulus Therapeutics.

Miravirsen (Roche/Santaris Pharma) is a phosphorothioate-modified LNA antagomiR, which targets miR-122 to repress hepatitis C viral (HCV) infections [180]. This drug entered phase 2 clinical trials in 2017. The miRNA regulatory mechanisms are vulnerable to attack from invading pathogens. Some viruses express miRNAs, which enter the endogenous silencing complex, to repress mRNAs that interfere with their replication [181]. Others, such as HCV, hijack specific cellular miRNAs and use it to its own advantage, rendering it essential for its own life cycle. HCV requires the action of miR-122, a liver-specific expressing miRNA [180,182,183], using it as a transcription factor to increase expression of the HCV genome, a mechanism essential for viral replication [184], making this an ideal candidate to inhibit the HCV infection. Miravirsen readily accumulates in the liver and as this is the site of action of the drug, does not require a specific delivery strategy. The outcome of the phase 2 clinical trials is positive, as patients who received miravirsen had undetectable HCV RNA levels [180], indicating that the replication of the virus was successfully repressed.

Another miR-122 targeting drug was developed by Regulus Therapeutics, RG-101. RG-101 is an N-acetyl-D-galactosamine-conjugated RNA antagomiR that targets miR-122 in HCV infected hepatocytes [185]. Like miravirsen, this drug showed great promise in lowering levels of HCV RNA to undetectable levels. However, due to adverse effects experienced by patients in clinical trials, further development of this drug, and other antagomiRs developed by the company, have been halted.

miRagen Therapeutics Inc. have a suite of miRNA antagomiRs in different drug development stages (Table 1), with one—MRG-106—showing promise treating some cancers. MRG-106, a LNA antagomiR that targets miR-155, is in phase 1 (NCT02580552) and phase 2 clinical trials (NCT03713320). miR-155 regulates differentiation and proliferation of blood and lymphoid cells and is a suitable target for treating certain kinds of lymphoma and leukaemia [186]. MRG-107 also targets miR-155 but has promise in treating patients with amyotrophic lateral sclerosis (ALS). However, this drug is not yet in clinical trials.

### 5.5. miRNA Mimic Drugs in Development

miRNA mimic drugs are also in development by miRagen Therapeutics Inc. (Table 1). Remlarsen (MRG-201), delivered by intradermal injection, is designed to mimic miR-29b, a negative regulator of extracellular matrix deposition, in order to decrease fibrous scar formation. miR-29b levels are decreased in fibrotic diseases and it is hoped that this drug will help patients with a predisposition to forming keloid scars. Remlarsen also has potential uses for pathological cutaneous fibrosis and idiopathic pulmonary fibrosis [187].

EnGeneIC Limited has successfully completed phase 1 clinical trials of a miRNA mimic drug. This drug is delivered as a TargomiR, encapsulated in a bacterial minicell. This particular TargomiR, MesomiR 1, mimics miR-16, which is targeted to Epidermal Growth Factor (EGFR)-expressing cancer cells via an EGFR-bispecific antibody and shows success treating malignant pleural mesothelioma and non-small-cell lung cancer [188]. Phase 2 clinical trials for mesomiR 1 are planned for the near future [189].

Unfortunately, severe reactions to some miRNA mimic drugs have been recorded in clinical trials, which is of great concern to the field. Mirna Therapeutics Inc. (Synlogic) developed a miRNA mimic for miR-34 (MRX34). Mir-34 functions as a tumour suppressor and is down regulated in a broad range of cancers [190,191,192]. MRX34 was delivered as a double-stranded RNA encapsulated in a liposome nanoparticle. While the drug showed promise in several preclinical studies in different cancers including renal cell carcinoma and hepatocellular carcinoma [193], clinical phase 1 trials were halted when immune-related serious adverse events resulting in death were reported. Consequently, all further development of the drug was halted.

## 6. Conclusions

RNA-based drugs are the latest frontier in nucleic acid therapeutics, with a considerable number in clinical trials. This demonstrates the significant potential of these therapeutic strategies, which promise to be effective in a wide range of currently untreatable disorders. The FDA approval of Onpattro is an exciting development and a significant boost to this field, and there is likely to be expansion in the breadth and scope of human disorders that can be treated using these approaches. 

Most oligonucleotide-based drugs are ASOs and siRNAs. However, there is potential that larger RNA species can be developed as therapeutics such as messenger RNA (mRNA). Compared to DNA-driven gene therapy, mRNA has distinct advantages: it can be effective in senescent cells, has higher penetrance rate in targeting cells and will not integrate into genome and risk developing mutation. Furthermore, in comparison to protein drugs, mRNA has a longer lifespan [194]. mRNA therapeutics are in clinical trials for metastatic prostate cancer [195]. Due to the length of mRNA, developing stable therapeutic molecules and efficient targeting delivery systems is needed for this field to expand [194]. mRNA drugs, however, may have a place in future replacement therapies.

Sequencing technologies have drastically improved the identification of new drug targets. However, a new tool, Clustered Regularly Interspersed Short Palindromic Repeats (CRISPR), is also promising to accelerate drug target identification [196]. The CRISPR complex is composed of a nuclease (e.g., CRISPR-associated protein 9 (Cas9)) and a guide RNA (gRNA) which specifically binds to and irreversibly cleaves complementary DNA sequences [197]. Synthesizing specific gRNA, will direct the complex to cleave desired regions of the genome and is a suitable tool for accurate target identification and replicating disease mutations. This tool could, therefore, rapidly identify new therapeutic targets [198]. Additionally, CRISPR’s ability to manipulate DNA could be directly used as a therapeutic and has shown potential in combating HIV and other infections [199]. CRISPR-based therapies are still in their infancy. However, even if they were approved for use in the future, RNA-based treatments have a distinct advantage—flexibility. As RNA-based treatments are delivered in repeated doses, this can be changed to suit the patient’s response and progress or discontinued if adverse reactions occur.

While ASOs and siRNA drugs appear to be gaining momentum in entering the clinic, miRNA drugs are yet to be successful. While a recent model tracking the growth of miRNA therapeutics indicated that the field had yet to mature [200], there may be physiological reasons for their current lack of success. miRNA-based regulation is an intricate network, with inbuilt redundancies [201] and few confirmed targets. It is probable that this network can absorb and ‘correct’ for the effect of a drug based on the regulatory action of one miRNA. It may be possible to overcome this by multiplexing miRNA therapies which target more than one member of a disease-associated pathway. Moreover, miRNA therapeutics may be most effective for treating pathogenic infections as there is no compensatory network in place.

Variations in genomic sequence, miRNA processing and miRNA turnover generate isomiRs (miRNA isoforms) [202]. IsomiRs are functionally important and have distinct roles in tissue-specific gene regulation [203]. Furthermore, isomiR expression changes in disease and when compared to canonical miRNAs, can better classify different tumour types [204,205]. Therefore, the specificity of diagnostic and prognostic panels could be considerably improved if isomiRs were also tested for their suitability as biomarkers.

A limitation of many current small molecule drugs is that the patients develop a resistance to the treatment, requiring higher and higher doses to be effective. This resistance to the drug corresponds to changes in miRNA expression, suggesting that miRNAs are involved in developing resistance or can influence the body’s response to the drug [206]. Co-administration of standard treatments with miRNA therapeutics may enhance drug bioavailability [207,208,209,210].

As novel therapeutics become more technology driven and personalised, there will be a corresponding increase in the cost of these treatments. For example, Nusinersen, the oligonucleotide-based drug to treat SMA, costs 708,000USD for the first year and 354,00USD for all subsequent years of treatment [211]. Therefore, there are many ethical implications to be considered to fully understand the social and health care requirements of the emerging new patient population that would benefit from this and similar drugs [212].

## Figures and Tables

**Table 1:** miRNA-based therapeutics in clinical trials.

miRNA-Based Therapeutics					
Company	Name	Therapeutic Agent	Delivery System	Target Disease	Stage in Drug Development Pipeline
Santaris Pharma/Roche	Miravirsen	AntimiR-122	LNA antagomiR	Hepatitis C; Chronic hepatitis C	Phase II clinical trials (NCT02452814; NCT2508090)
Regulus Therapeutics	RG-101	AntimiR-122	GaLNAc-conjugated antagomiR	Chronic hepatitis C	Phase II clinical trials (discontinued)
	RG-125	AntimiR-103/107	GaLNAc-conjugated antagomiR	Diabetic non-alcoholic steatohepatitis	Phase II (discontinued)
	RG-012	AntimiR-21	NA	Hereditary nephritis	Phase II (NCT02855268)
	RGLS4326	AntimiR-17	NA	Autosomal dominant polycystic kidney disease	Phase I (on hold)
miRagen Therapeutics	MRG-106	AntimiR-155	LNA-modified antisense inhibitor	CTCL mycosis fungoides subtype; CLL; DLBCL; ATLL	Phase II (NCT03713320; NCT03837457); Phase I (NCT02580552)
	MRG-107	AntimiR-155	NA	ALS; cardiac disorders; retinal disorders	Pre-Clinical
	MRG-110	AntimiR-92	LNA antagomiR	Wounds	Phase I (NCT03603431)
	MRG-201	miR-29 mimic	Cholesterol-conjugated miRNA duplex	Keloid; fibrosis	Phase II (NCT03601052); Phase I (NCT02603224)
EnGeneIC	MesomiR-1	miR-16 mimic	EnGeneIC Dream Vector	Malignant pleural mesothelioma; non-small-cell lung cancer	Phase I (NCT02369198)
Mirna Therapeutics Inc.	MRX-34	miR-34 mimic	dsRNA liposomal nanoparticle	Solid tumours; haematological malignancies	Phase 1 (terminated)

LNA: locked nucleic acid; GaLNAc: N-acetylgalactosamine; CTCL: cutaneous T cell lymphoma; ATLL: adult T-cell leukaemia lymphoma; CLL: chronic lymphocytic leukaemia; DLBCL: diffuse large B-cell lymphoma [activated B-cell (ABC) subtype]; ALS: amyloid lateral sclerosis.

## References

[1] SoRelle R. (2000). Who Owns you DNA? Who Will own it?. Circulation.

[2] Anderson W.F. (1998). Human gene therapy. Nature.

[3] Otsu M., Candotti F. (2002). Gene therapy in infants with severe combined immunodeficiency. BioDrugs.

[4] Baekelandt V., De Strooper B., Nuttin B., Debyser Z. (2000). Gene therapeutic strategies for neurodegenerative diseases. Curr. Opin. Mol. Ther..

[5] Galanis E., Russell S. (2001). Cancer gene therapy clinical trials: Lessons for the future. Br. J. Cancer.

[6] Saraswat P., Soni R.R., Bhandari A., Nagori B.P. (2009). DNA as therapeutics; an update. Indian J. Pharm. Sci..

[7] Myhr A.I. (2007). DNA Vaccines: Regulatory Considerations and Safety Aspects. Curr. Issues Mol. Biol..

[8] Perry C.M., Balfour J.A. (1999). Fomivirsen. Drugs.

[9] Grillone L.R., Lanz R. (2001). Fomivirsen. Drugs Today.

[10] Liu B., Montgomery S.B. (2019). Identifying causal variants and genes using functional genomics in specialized cell types and contexts. Hum. Genet..

[11] Matsui M., Corey D.R. (2017). Non-coding RNAs as drug targets. Nat. Rev. Drug Discov..

[12] Rupaimoole R., Slack F.J. (2017). MicroRNA therapeutics: Towards a new era for the management of cancer and other diseases. Nat. Rev. Drug Discov..

[13] Harries L.W. (2019). RNA biology provides new therapeutic targets for human disease. Front. Genet..

[14] Lee R., Feinbaum R., Ambros V. (1993). The C. elegans Heterochronic Gene lin-4 Encodes Small RNAs with Antisense Complementarity to lin-14. Cell.

[15] Olsen P.H., Ambros V. (1999). The lin-4 regulatory RNA controls developmental timing in Caenorhabditis elegans by blocking LIN-14 protein synthesis after the initiation of translation. Dev. Biol..

[16] Wilson R., Doudna J.A. (2013). Molecular mechanisms of RNA interference A BIOLOGICAL VIEW OF RNA INTERFERENCE • Small regulatory RNAs in cellular function and dysfunction HHS Public Access. Annu. Rev. Biophys..

[17] Bartel D.P. (2009). MicroRNAs: Target recognition and regulatory functions. Cell.

[18] Hammond S.M. (2015). An overview of microRNAs Scott. Adv. Drug Deliv. Rev..

[19] Friedman R.C., Farh K.K.-H., Burge C.B., Bartel D.P. (2009). Most mammalian mRNAs are conserved targets of microRNAs. Genome Res..

[20] Bajan S., Hutvagner G. (2014). Regulation of miRNA Processing and miRNA Mediated Gene Repression in Cancer. MicroRNA.

[21] Gorabi A.M., Bianconi V., Pirro M., Banach M. (2019). Biomedicine & Pharmacotherapy Regulation of cardiac stem cells by microRNAs: State-of-the-art. Biomed. Pharmacother..

[22] Yang Y., Luo C. (2019). MicroRNAs in acute pancreatitis: From pathogenesis to novel diagnosis and therapy. J. Cell. Physiol..

[23] Hrach H.C., Mangone M. (2019). miRNA Profiling for Early Detection and Treatment of Duchenne Muscular Dystrophy. Int. J. Mol. Sci..

[24] Evangelatos G., Fragoulis G.E., Koulouri V., Lambrou G.I. (2019). Micrornas in rheumatoid arthritis: From pathogenesis to clinical impact. Autoimmun. Rev..

[25] Bonneau E., Neveu B., Kostantin E., Tsongalis G.J., De Guire V. (2019). How close are miRNAs from clinical practice? A perspective on the diagnostic and therapeutic market. Ejifcc.

[26] Rani A., O’Shea A., Ianov L., Cohen R.A., Woods A.J., Foster T.C. (2017). miRNA in circulating microvesicles as biomarkers for age-related cognitive decline. Front. Aging Neurosci..

[27] Adams B.D., Parsons C., Walker L., Zhang W.C., Slack F.J. (2017). Targeting noncoding RNAs in disease. J. Clin. Invest..

[28] Blake S.J., Bokhari F.F., McMillan N.A. (2012). RNA Interference for Viral Infections. Curr. Drug Targets.

[29] Rossor A.M., Reilly M.M., Sleigh J.N. (2018). Antisense oligonucleotides and other genetic therapies made simple. Pract. Neurol..

[30] Bennett C.F., Baker B.F., Pham N., Swayze E., Geary R.S. (2017). Pharmacology of Antisense Drugs. Annu. Rev. Pharmacol. Toxicol..

[31] Schuster S., Miesen P., van Rij R.P. (2019). Antiviral RNAi in insects and mammals: Parallels and differences. Viruses.

[32] Sharp P.A. (1999). RNAi and double-strand RNA. Genes. Dev..

[33] Schwarz D.S., Ding H., Kennington L., Moore J.T., Schelter J., Burchard J., Linsley P.S., Aronin N., Xu Z., Zamore P.D. (2006). Designing siRNA that distinguish between genes that differ by a single nucleotide. PLoS Genet..

[34] Jackson A.L., Linsley P.S. (2010). Recognizing and avoiding siRNA off-target effects for target identification and therapeutic application. Nat. Rev. Drug Discov..

[35] Grimm D. (2011). The dose can make the poison: Lessons learned from adverse in vivo toxicities caused by RNAi overexpression. Silence.

[36] Grimm D., Streetz K.L., Jopling C.L., Storm T.A., Pandey K., Davis C.R., Marion P., Salazar F., Kay M.A. (2006). Fatality in mice due to oversaturation of cellular microRNA/short hairpin RNA pathways. Nature.

[37] Chakraborty C., Sharma A.R., Sharma G., Doss C.G.P., Lee S.S. (2017). Therapeutic miRNA and siRNA: Moving from Bench to Clinic as Next Generation Medicine. Mol. Ther. Nucl. Acid..

[38] Liu Y.P., Haasnoot J., Berkhout B. (2007). Design of extended short hairpin RNAs for HIV-1 inhibition. Nucl. Acid. Res..

[39] Liu Y.P., von Eije K.J., Schopman N.C.T., Westerink J.T., ter Brake O., Haasnoot J., Berkhout B. (2009). Combinatorial RNAi against HIV-1 using extended short hairpin RNAs. Mol. Ther..

[40] Kaczmarek J.C., Kowalski P.S., Anderson D.G. (2017). Advances in the delivery of RNA therapeutics: From concept to clinical reality. Genome Med..

[41] Haussecker D. (2014). Current issues of RNAi therapeutics delivery and development. J. Control. Release.

[42] Shukla S., Sumaria C.S., Pradeepkumar P.I. (2010). Exploring chemical modifications for siRNA therapeutics: A structural and functional outlook. ChemMedChem.

[43] Egli M., Manoharan M. (2019). Re-Engineering RNA Molecules into Therapeutic Agents. Acc. Chem. Res..

[44] Judge A.D., Bola G., Lee A.C.H., MacLachlan I. (2006). Design of noninflammatory synthetic siRNA mediating potent gene silencing in vivo. Mol. Ther..

[45] Jackson A.L., Burchard J., Schelter J., Chau B.N., Cleary M., Lim L., Linsley P.S. (2006). Widespread siRNA “off-target” transcript silencing mediated by seed region sequence complementarity. RNA.

[46] Davis S., Lollo B., Freier S., Esau C. (2006). Improved targeting of miRNA with antisense oligonucleotides. Nucleic Acids Res..

[47] Esau C., Davis S., Murray S.F., Yu X.X., Pandey S.K., Pear M., Watts L., Booten S.L., Graham M., McKay R. (2006). miR-122 regulation of lipid metabolism revealed by in vivo antisense targeting. Cell Metab..

[48] Ørom U.A., Kauppinen S., Lund A.H. (2006). LNA-modified oligonucleotides mediate specific inhibition of microRNA function. Gene.

[49] Lennox K.A., Behlke M.A. (2010). A direct comparison of anti-microRNA oligonucleotide potency. Pharm. Res..

[50] Elmén J., Lindow M., Schütz S., Lawrence M., Petri A., Obad S., Lindholm M., Hedtjärn M., Hansen H.F., Berger U. (2008). LNA-mediated microRNA silencing in non-human primates. Nature.

[51] Lanford R.E., Hildebrandt-Eriksen E.S., Petri A., Persson R., Lindow M., Munk M.E., Kauppinen S., Ørum H. (2010). Therapeutic silencing of microRNA-122 in primates with chronic hepatitis C virus infection. Science..

[52] Soutschek J., Akinc A., Bramlage B., Charisse K., Constien R., Donoghue M., Elbashir S., Gelck A., Hadwiger P., Harborth J. (2004). Therapeutic silencing of an endogenous gene by systemic administration of modified siRNAs. Nature.

[53] Wolfrum C., Shi S., Jayaprakash K.N., Jayaraman M., Wang G., Pandey R.K., Rajeev K.G., Nakayama T., Charrise K., Ndungo E.M. (2007). Mechanisms and optimization of in vivo delivery of lipophilic siRNAs. Nat. Biotechnol..

[54] Petrova N.S., Chernikov I.V., Meschaninova M.I., Dovydenko I.S., Venyaminova A.G., Zenkova M.A., Vlassov V.V., Chernolovskaya E.L. (2012). Carrier-free cellular uptake and the gene-silencing activity of the lipophilic siRNAs is strongly affected by the length of the linker between siRNA and lipophilic group. Nucleic Acids Res..

[55] Letsinger R.L., Zhang G., Sun D.K., Ikeuchi T., Sarin P.S. (1989). Cholesteryl-conjugated oligonucleotides: Synthesis, properties, and activity as inhibitors of replication of human immunodeficiency virus in cell culture. Proc. Natl. Acad. Sci. USA.

[56] Burnett J.C., Rossi J.J. (2012). RNA-based therapeutics: Current progress and future prospects. Chem. Biol..

[57] Peer D., Lieberman J. (2011). Special delivery: Targeted therapy with small RNAs. Gene Ther..

[58] Chiriboga C.A. (2017). Expert Review of Neurotherapeutics Nusinersen for the treatment of spinal muscular atrophy Nusinersen for the treatment of spinal muscular atrophy. Expert Rev. Neurother..

[59] Lares M.R., Rossi J.J., Ouellet D.L. (2010). RNAi and small interfering RNAs in human disease therapeutic applications. Trends Biotechnol..

[60] Ligtenberg M.A., Pico de Coaña Y., Shmushkovich T., Yoshimoto Y., Truxova I., Yang Y., Betancur-Boissel M., Eliseev A.V., Wolfson A.D., Kiessling R. (2018). Self-Delivering RNAi Targeting PD-1 Improves Tumor-Specific T Cell Functionality for Adoptive Cell Therapy of Malignant Melanoma. Mol. Ther..

[61] McNamara J.O., Andrechek E.R., Wang Y., Viles K.D., Rempel R.E., Gilboa E., Sullenger B.A., Giangrande P.H. (2006). Cell type-specific delivery of siRNAs with aptamer-siRNA chimeras. Nat. Biotechnol..

[62] Dassie J.P., Liu X.Y., Thomas G.S., Whitaker R.M., Thiel K.W., Stockdale K.R., Meyerholz D.K., McCaffrey A.P., McNamara J.O., Giangrande P.H. (2009). Systemic administration of optimized aptamer-siRNA chimeras promotes regression of PSMA-expressing tumors. Nat. Biotechnol..

[63] Zhou J., Swiderski P., Li H., Zhang J., Neff C.P., Akkina R., Rossi J.J. (2009). Selection, characterization and application of new RNA HIV gp 120 aptamers for facile delivery of Dicer substrate siRNAs into HIV infected cells. Nucleic Acids Res..

[64] Kumar P., Ban H.S., Kim S.S., Wu H., Pearson T., Greiner D.L., Laouar A., Yao J., Haridas V., Habiro K. (2008). T Cell-Specific siRNA Delivery Suppresses HIV-1 Infection in Humanized Mice. Cell.

[65] Song E., Zhu P., Lee S.K., Chowdhury D., Kussman S., Dykxhoorn D.M., Feng Y., Palliser D., Weiner D.B., Shankar P. (2005). Antibody mediated in vivo delivery of small interfering RNAs via cell-surface receptors. Nat. Biotechnol..

[66] Peer D., Park E.J., Morishita Y., Carman C.V., Shimaoka M. (2008). Systemic Leukocyte-Directed siRNA Delivery Revealing Cyclin D1 as an Anti-Inflammatory Target. Science.

[67] Morrissey D.V., Lockridge J.A., Shaw L., Blanchard K., Jensen K., Breen W., Hartsough K., Machemer L., Radka S., Jadhav V. (2005). Potent and persistent in vivo anti-HBV activity of chemically modified siRNAs. Nat. Biotechnol..

[68] Frank-Kamenetsky M., Grefhorst A., Anderson N.N., Racie T.S., Bramlage B., Akinc A., Butler D., Charisse K., Dorkin R., Fan Y. (2008). Therapeutic RNAi targeting PCSK9 acutely lowers plasma cholesterol in rodents and LDL cholesterol in nonhuman primates. Proc. Natl. Acad. Sci. USA.

[69] Sonoke S., Ueda T., Fujiwara K., Sato Y., Takagaki K., Hirabayashi K., Ohgi T., Yano J. (2008). Tumor regression in mice by delivery of Bcl-2 small interfering RNA with pegylated cationic liposomes. Cancer Res..

[70] Azuma K., Nakashiro K.I., Sasaki T., Goda H., Onodera J., Tanji N., Yokoyama M., Hamakawa H. (2010). Anti-tumor effect of small interfering RNA targeting the androgen receptor in human androgen-independent prostate cancer cells. Biochem. Biophys. Res. Commun..

[71] Wu Y., Wang W., Chen Y., Huang K., Shuai X., Chen Q., Li X., Lian G. (2010). The investigation of polymer-siRNA nanoparticle for gene therapy of gastric cancer in vitro. Int. J. Nanomed..

[72] Sun H.K., Ji H.J., Kim T.I., Sung W.K., Bull D.A. (2009). VEGF siRNA delivery system using arginine-grafted bioreducible poly(disulfide amine). Mol. Pharm..

[73] Yam P.Y., Li S., Wu J., Hu J., Zaia J.A., Yee J.K. (2002). Design of HIV vectors for efficient gene delivery into human hematopoietic cells. Mol. Ther..

[74] Miyoshi H., Blömer U., Takahashi M., Gage F.H., Verma I.M. (1998). Development of a self-inactivating lentivirus vector. J. Virol..

[75] VandenDriessche T., Thorrez L., Naldini L., Follenzi A., Moons L., Berneman Z., Collen D., Chuah M.K.L. (2002). Lentiviral vectors containing the human immunodeficiency virus type-1 central polypurine tract can efficiently transduce nondividing hepatocytes and antigen-presenting cells in vivo. Blood.

[76] Hacein-Bey-Abina S., Von Kalle C., Schmidt M., McCormack M.P., Wulffraat N., Leboulch P., Lim A., Osborne C.S., Pawliuk R., Morillon E. (2003). LMO2- Associated Clocal T cell Prolferation in Two Patients after Gene Therapy for SCID-X1. Science.

[77] Gijsbers R., Ronen K., Vets S., Malani N., De Rijck J., McNeely M., Bushman F.D., Debyser Z. (2010). LEDGF hybrids efficiently retarget lentiviral integration into heterochromatin. Mol. Ther..

[78] Michelfelder S., Trepel M. (2009). Chapter 2-Adeno-Associated Viral Vectors and Their Redirection to Cell-Type Specific Receptors. Tissue-Specific Vascular Endothelial Signals and Vector Targeting, Part A.

[79] MacDiarmid J.A., Brahmbhatt H. (2011). Minicells: Versatile vectors for targeted drug or si/shRNA cancer therapy. Curr. Opin. Biotechnol..

[80] MacDiarmid J.A., Mugridge N.B., Weiss J.C., Phillips L., Burn A.L., Paulin R.P.P., Haasdyk J.E., Dickson K.A., Brahmbhatt V.N., Pattison S.T. (2007). Bacterially Derived 400 nm Particles for Encapsulation and Cancer Cell Targeting of Chemotherapeutics. Cancer Cell.

[81] MacDiarmid J.A., Amaro-Mugridge N.B., Madrid-Weiss J., Sedliarou I., Wetzel S., Kochar K., Brahmbhatt V.N., Phillips L., Pattison S.T., Petti C. (2009). Sequential treatment of drug-resistant tumors with targeted minicells containing siRNA or a cytotoxic drug. Nat. Biotechnol..

[82] Akira S., Takeda K. (2004). Toll-like receptor signalling. Nat. Rev. Immunol..

[83] Marques J.T., Williams B.R.G. (2005). Activation of the mammalian immune system by siRNAs. Nat. Biotechnol..

[84] Poeck H., Besch R., Maihoefer C., Renn M., Tormo D., Morskaya S.S., Kirschnek S., Gaffal E., Landsberg J., Hellmuth J. (2008). 5′-triphosphate-siRNA: Turning gene silencing and Rig-I activation against melanoma. Nat. Med..

[85] Gantier M.P., Tong S., Behlke M.A., Irving A.T., Lappas M., Nilsson U.W., Latz E., McMillan N.A.J., Williams B.R.G. (2010). Rational design of immunostimulatory siRNAs. Mol. Ther..

[86] Sugarman E.A., Nagan N., Zhu H., Akmaev V.R., Zhou Z., Rohlfs E.M., Flynn K., Hendrickson B.C., Scholl T., Sirko-Osadsa D.A. (2012). Pan-ethnic carrier screening and prenatal diagnosis for spinal muscular atrophy: Clinical laboratory analysis of >72 400 specimens. Eur. J. Hum. Genet..

[87] Lorson C.L., Hahnen E., Androphy E.J., Wirth B. (1999). A single nucleotide in the SMN gene regulates splicing and is responsible for spinal muscular atrophy. Proc. Natl. Acad. Sci. USA.

[88] Monani U.R., Lorson C.L., Parsons D.W., Prior T.W., Androphy E.J., Burghes A.H.M., McPherson J.D. (1999). A single nucleotide difference that alters splicing patterns distinguishes the SMA gene SMN1 from the copy gene SMN2. Hum. Mol. Genet..

[89] Mailman M.D., Heinz J.W., Papp A.C., Snyder P.J., Sedra M.S., Wirth B., Burghes A.H.M., Prior T.W. (2002). Molecular analysis of spinal muscular atrophy and modification of the phenotype by SMN2. Genet. Med..

[90] Wirth B., Herz M., Wetter A., Moskau S., Hahnen E., Wienker T., Zerres K. (1999). Quantitative Analysis of Survival Motor Neuron Copies: Identification of Subtle SMN1 Mutations in Patients with Spinal Muscular Atrophy, Genotype-Phenotype Correlation and Implications for Genetic Counseling. Am. J. Hum. Genet..

[91] FDA U.S. (2017). Department of Health and Human Services, Center for Drug Evaluation and Research. Application Number: 209531Orig1s000. Med. Rev..

[92] Kole R., Krieg A.M. (2015). Exon skipping therapy for Duchenne muscular dystrophy. Adv. Drug Deliv. Rev..

[93] Singh N.K., Singh N.K., Singh R.N., Singh R.N. (2006). Splicing of a Critical Exon of Human *Survival Motor Neuron* is Regulated by a Unique Silencer Element Located in the Last Intron. Mol. Cell. Biol..

[94] Carpenter S., Karpati G. (1979). Duchenne Muscular Dystophy: Plasma Membrane Loss Initiates Muscle Cell Necrosis Unless it is Repaired. Brain.

[95] Emery A.E.H. (2002). The muscular dystrophies. Lancet.

[96] Van Deutekom J.C., Janson A.A., Ginjaar I.B., Frankhuizen W.S., Aartsma-Rus A., Bremmer-Bout M., Den Dunnen J.T., Koop K., Van Der Kooi A.J., Goemans N.M. (2007). Local dystrophin restoration with antisense oligonucleotide PRO051. N. Engl. J. Med..

[97] Goemans N.M., Tulinius M., Van Den Akker J.T., Burm B.E., Ekhart P.F., Heuvelmans N., Holling T., Janson A.A., Platenburg G.J., Sipkens J.A. (2011). Systemic administration of PRO051 in Duchenne’s muscular dystrophy. N. Engl. J. Med..

[98] Aartsma-Rus A., Straub V., Hemmings R., Haas M., Schlosser-Weber G., Stoyanova-Beninska V., Mercuri E., Muntoni F., Sepodes B., Vroom E. (2017). Development of Exon Skipping Therapies for Duchenne Muscular Dystrophy: A Critical Review and a Perspective on the Outstanding Issues. Nucleic Acid Ther..

[99] Aartsma-Rus A., Krieg A.M. (2017). FDA Approves Eteplirsen for Duchenne Muscular Dystrophy: The Next Chapter in the Eteplirsen Saga. Nucleic Acid Ther..

[100] FDA (2018). ONPATTRO (patisiran) lipid complex injection, for intravenous use. Highlights Prescrib. Inf..

[101] European Medicines Agency (2018). Onpattro (Patisiran): An Overview of Onpattro and Why It Is Authorised in the EU.

[102] Kristen A.V., Ajroud-Driss S., Conceição I., Gorevic P., Kyriakides T., Obici L. (2019). Patisiran, an RNAi therapeutic for the treatment of hereditary transthyretin-mediated amyloidosis. Neurodegener. Dis. Manag..

[103] Yang J. (2019). Patisiran for the treatment of hereditary transthyretin-mediated amyloidosis. Expert Rev. Clin. Pharmacol..

[104] Plante-Bordeneuve V., Said G. (2011). Familial amyloid polyneuropathy. Lancet Neurol.

[105] Waddington-Cruz M., Ackermann E.J., Polydefkis M., Heitner S.B., Dyck P.J., Barroso F.A., Wang A.K., Berk J.L., Dyck P.J.B., Monia B.P. (2018). Hereditary transthyretin amyloidosis: Baseline characteristics of patients in the NEURO-TTR trial. Amyloid.

[106] Lopes A., Rodrigues C., Fonseca I., Sousa A., Branco M., Coelho T., Sequeiros J., Freitas P. (2018). Family dynamics in transthyretin-related familial amyloid polyneuropathy Val30Met: Does genetic risk affect family functioning?. Clin. Genet..

[107] Hawkins P.N., Ando Y., Dispenzeri A., Gonzalez-Duarte A., Adams D., Suhr O.B. (2015). Evolving landscape in the management of transthyretin amyloidosis. Ann. Med..

[108] Buxbaum J.N. (2018). Oligonucleotide Drugs for Transthyretin Amyloidosis. N. Engl. J. Med..

[109] Maia L.F., Magalhães R., Freitas J., Taipa R., Pires M.M., Osório H., Dias D., Pessegueiro H., Correia M., Coelho T. (2015). CNS involvement in V30M transthyretin amyloidosis: Clinical, neuropathological and biochemical findings. J. Neurol. Neurosurg. Psychiatry.

[110] Lavigne-Moreira C., Marques V.D., Gonçalves M.V.M., de Oliveira M.F., Tomaselli P.J., Nunez J.C., do Nascimento O.J.M., Barreira A.A., Marques W. (2018). The genetic heterogeneity of hereditary transthyretin amyloidosis in a sample of the Brazilian population. J. Peripher. Nerv. Syst..

[111] Coelho T., Adams D., Silva A., Lozeron P., Hawkins P.N., Mant T., Perez J., Chiesa J., Warrington S., Tranter E. (2013). Safety and efficacy of RNAi therapy for transthyretin amyloidosis. N. Engl. J. Med..

[112] Walker S. (2018). New Medications in the Treatment of Hereditary Transthyretin Amyloidosis. Hosp. Pharm..

[113] Suhr O.B., Coelho T., Buades J., Pouget J., Conceicao I., Berk J., Schmidt H., Waddington-Cruz M., Campistol J.M., Bettencourt B.R. (2015). Efficacy and safety of patisiran for familial amyloidotic polyneuropathy: A phase II multi-dose study. Orphanet J. Rare Dis..

[114] Adams D., Coelho T., Conceicao E., Waddington-Cruz M., Schmidt H., Buades J., Campistol J., Pouget J., Berk J., Polydefkis M. (2017). Phase 2 Open-Label Extension (Ole) Study of Patisiran, an Investigational Rna Interference (Rnai) Therapeutic for the Treatment of Hereditary Attr Amyloidosis with Polyneuropathy. Value Heal..

[115] European Medicines Agency (2018). Summary of Product Characteristics: Tegsedi 284mg Solution for Injection in Pre-Filled Syringe.

[116] Akcea Therapeutics Inc (2018). US Prescribing Information: TEGSEDI^TM^ (Inotersen) Injection, for Subcutaneous Use.

[117] Aboul-Fadl T. (2005). Antisense Oligonucleotides: The State of the Art. Curr. Med. Chem..

[118] Elbashir S.M., Harborth J., Lendeckel W., Yalcin A., Weber K., Tuschl T. (2001). Duplexes of 21-nucleotide RNAs mediate RNA interference in cultured mammalian cells. Nature.

[119] Garba A.O., Mousa S.A. (2010). Bevasiranib for the Treatment of Wet, Age-Related Macular Degeneration. Ophthalmol. Eye Dis..

[120] Singerman L. (2009). Combination therapy using the small interfering RNA bevasiranib. Retina.

[121] Zhang K., Zhang L., Weinreb R.N. (2012). Ophthalmic drug discovery: Novel targets and mechanisms for retinal diseases and glaucoma. Nat. Rev. Drug Discov..

[122] Worringer K.A., Rand T.A., Hayashi Y., Sami S., Takahashi K., Tanabe K., Narita M., Srivastava D., Yamanaka S. (2014). The let-7/LIN-41 pathway regulates reprogramming to human induced pluripotent stem cells by controlling expression of prodifferentiation genes. Cell Stem Cell.

[123] Reinhart B.J., Slack F.J., Basson M., Pasquinelli A.E., Bettinger J.C., Rougvie A.E., Horvitz H.R., Ruvkun G. (2000). The 21-nucleotide let-7 RNA regulates developmental timing in Caenorhabditis elegans. Nat..

[124] Bartel D.P. (2004). MicroRNAs: Genomics, Biogenesis, Mechanism, and Function. Cell.

[125] Rupaimoole R., Calin G.A., Lopez-Berestein G., Sood A.K. (2016). MiRNA deregulation in cancer cells and the tumor microenvironment. Cancer Discov..

[126] Ha M., Kim V.N. (2014). Regulation of microRNA biogenesis. Nat. Rev. Mol. Cell Biol..

[127] Lin S., Gregory R.I. (2015). MicroRNA biogenesis pathways in cancer. Nat. Rev. Cancer.

[128] Wang J., Lu Z., Wientjes M.G., Au J.L.S. (2010). Delivery of siRNA therapeutics: Barriers and carriers. AAPS J..

[129] Li Z., Rana T.M. (2014). Therapeutic targeting of microRNAs: Current status and future challenges. Nat. Rev. Drug Discov..

[130] Zhang S., Yue W., Xie Y., Liu L., Li S., Dang W., Xin S., Yang L., Zhai X., Cao P. (2019). The four-microRNA signature identified by bioinformatics analysis predicts the prognosis of nasopharyngeal carcinoma patients. Oncol. Rep..

[131] Andersen G.B., Tost J., Schaffner F., Merlin J.L., von Bubnoff N. (2020). Circulating miRNAs as Biomarker in Cancer. Tumor Liquid Biopsies.

[132] Cavalcante P., Mizrachi T., Barzago C., Scandiffio L., Bortone F., Bonanno S., Frangiamore R., Mantegazza R., Bernasconi P., Brenner T. (2019). MicroRNA signature associated with treatment response in myasthenia gravis: A further step towards precision medicine. Pharmacol. Res..

[133] Wang Y., Ru J., Jin T., Sun M., Jia L., Sun G. (2019). An Approach to Identify Individual Functional Single Nucleotide Polymorphisms and Isoform MicroRNAs. Biomed. Res. Int..

[134] Rooij E., Kauppinen S. (2014). Development of micro RNA therapeutics is coming of age. EMBO Mol. Med..

[135] Jazbutyte V. (2012). Specific, Robust, Reproducible: The Hunt for the Ideal Biomarker. J. Clin. Exp. Cardiol..

[136] De Guire V., Robitaille R., Tétreault N., Guérin R., Ménard C., Bambace N., Sapieha P. (2013). Circulating miRNAs as sensitive and specific biomarkers for the diagnosis and monitoring of human diseases: Promises and challenges. Clin. Biochem..

[137] Mitchell P.S., Parkin R.K., Kroh E.M., Fritz B.R., Wyman S.K., Pogosova-Agadjanyan E.L., Peterson A., Noteboom J., O’Briant K.C., Allen A. (2008). Circulating microRNAs as stable blood-based markers for cancer detection. Proc. Natl. Acad. Sci. USA.

[138] Chen X., Ba Y., Ma L., Cai X., Yin Y., Wang K., Guo J., Zhang Y., Chen J., Guo X. (2008). Characterization of microRNAs in serum: A novel class of biomarkers for diagnosis of cancer and other diseases. Cell Res..

[139] Weber J.A., Baxter D.H., Zhang S., Huang D.Y., Huang K.H., Lee M.J., Galas D.J., Wang K. (2010). The microRNA spectrum in 12 body fluids. Clin. Chem..

[140] Landgraf P., Rusu M., Sheridan R., Sewer A., Iovino N., Aravin A., Pfeffer S., Rice A., Kamphorst A.O., Landthaler M. (2007). A Mammalian microRNA Expression Atlas Based on Small RNA Library Sequencing. Cell.

[141] Jung M., Schaefer A., Steiner I., Kempkensteffen C., Stephan C., Erbersdobler A., Jung K. (2010). Robust MicroRNA stability in degraded RNA preparations from human tissue and cell samples. Clin. Chem..

[142] Kumar M.S., Erkeland S.J., Pester R.E., Chen C.Y., Ebert M.S., Sharp P.A., Jacks T. (2008). Suppression of non-small cell lung tumor development by the let-7 microRNA family. Proc. Natl. Acad. Sci. USA.

[143] Gustafson D., Tyryshkin K., Renwick N. (2016). microRNA-guided diagnostics in clinical samples. Best Pract. Res. Clin. Endocrinol. Metab..

[144] Kim Y.K., Yeo J., Kim B., Ha M., Kim V.N. (2012). Short Structured RNAs with Low GC Content Are Selectively Lost during Extraction from a Small Number of Cells. Mol. Cell.

[145] Leichter A.L., Purcell R.V., Sullivan M.J., Eccles M.R., Chatterjee A. (2015). Multi-platform microRNA profiling of hepatoblastoma patients using formalin fixed paraffin embedded archival samples. Gigascience.

[146] Git A., Dvinge H., Salmon-Divon M., Osborne M., Kutter C., Hadfield J., Bertone P., Caldas C. (2001). Systematic comparison of microarray profiling, real-time PCR, and next-generation sequencing technologies for measuring differential microRNA expression. RNA.

[147] Sato F., Tsuchiya S., Terasawa K., Tsujimoto G. (2009). Intra-platform repeatability and inter-platform comparability of microRNA microarray technology. PLoS ONE.

[148] Qin L.X., Tuschl T., Singer S. (2016). Empirical insights into the stochasticity of small RNA sequencing. Sci. Rep..

[149] Yu H., Guan Z., Cuk K., Brenner H., Zhang Y. (2018). Circulating microRNA biomarkers for lung cancer detection in Western populations. Cancer Med..

[150] Rawlings-Goss R.A., Campbell M.C., Tishkoff S.A. (2014). Global population-specific variation in miRNA associated with cancer risk and clinical biomarkers. BMC Med. Genomics.

[151] Rissin D.M., López-Longarela B., Pernagallo S., Ilyine H., Vliegenthart A.D.B., Dear J.W., Díaz-Mochón J.J., Duffy D.C. (2017). Polymerase-free measurement of microRNA-122 with single base specificity using single molecule arrays: Detection of drug-induced liver injury. PLoS ONE.

[152] Proschak E., Stark H., Merk D. (2019). Polypharmacology by Design: A Medicinal Chemist’s Perspective on Multitargeting Compounds. J. Med. Chem..

[153] Boran A., Iyengar R. (2010). Systems approaches to polypharmacology and drug discovery. Curr. Opin Drug Discov. Dev..

[154] Lim L.P., Lau N.C., Garrett-engele P., Grimson A. (2005). Microarray analysis shows that some microRNAs downregulate large numbers of target mRNAs. Nature.

[155] Singh S., Narang A.S., Mahato R.I. (2011). Subcellular fate and off-target effects of siRNA, shRNA, and miRNA. Pharm. Res..

[156] Chen Y., Zhao H., Tan Z., Zhang C., Fu X. (2015). Bottleneck limitations for microRNA-based therapeutics from bench to the bedside. Pharmazie.

[157] Lal A., Navarro F., Maher C.A., Maliszewski L.E., Yan N., O’Day E., Chowdhury D., Dykxhoorn D.M., Tsai P., Hofmann O. (2009). miR-24 Inhibits Cell Proliferation by Targeting E2F2, MYC, and Other Cell-Cycle Genes via Binding to “Seedless” 3′UTR MicroRNA Recognition Elements. Mol. Cell.

[158] Seitz H. (2009). Redefining MicroRNA Targets. Curr. Biol..

[159] Lee Y.J., Kim V., Muth D.C., Witwer K.W. (2015). Validated MicroRNA Target Databases: An Evaluation. Drug Dev. Res..

[160] Leclercq M., Diallo A.B., Blanchette M. (2017). Prediction of human miRNA target genes using computationally reconstructed ancestral mammalian sequences. Nucleic Acids Res..

[161] Metias S.M., Lianidou E., Yousef G.M. (2009). MicroRNAs in clinical oncology: At the crossroads between promises and problems. J. Clin. Pathol..

[162] Farooqi A.A., Fayyaz S., Shatynska-Mytsyk I., Javed Z., Jabeen S., Yaylim I., Gasparri M.L., Panici P.B. (2016). Is miR-34a a Well-equipped Swordsman to Conquer Temple of Molecular Oncology?. Chem. Biol. Drug Des..

[163] Esquela-Kerscher A., Trang P., Wiggins J.F., Patrawala L., Cheng A., Ford L., Weidhaas J.B., Brown D., Bader A.G., Slack F.J. (2008). The let-7 microRNA reduces tumor growth in mouse models of lung cancer. Cell Cycle.

[164] Bonci D., Coppola V., Musumeci M., Addario A., Giuffrida R., Memeo L., D’Urso L., Pagliuca A., Biffoni M., Labbaye C. (2008). The miR-15a-miR-16-1 cluster controls prostate cancer by targeting multiple oncogenic activities. Nat. Med..

[165] Boudreau R.L., Monteys A.M., Davidson B.L. (2008). Minimizing variables among hairpin-based RNAi vectors reveals the potency of shRNAs. RNA.

[166] Bauer M., Kinkl N., Meixner A., Kremmer E., Riemenschneider M., Förstl H., Gasser T., Ueffing M. (2009). Prevention of interferon-stimulated gene expression using microRNA-designed hairpins. Gene Ther..

[167] Aagaard L.A., Zhang J., von Eije K.J., Li H., Sætrom P., Amarzguioui M., Rossi J.J. (2008). Engineering and optimization of the miR-106b cluster for ectopic expression of multiplexed anti-HIV RNAs. Gene Ther..

[168] Liu Y.P., Haasnoot J., ter Brake O., Berkhout B., Konstantinova P. (2008). Inhibition of HIV-1 by multiple siRNAs expressed from a single microRNA polycistron. Nucleic Acids Res..

[169] Johnson C.D., Esquela-Kerscher A., Stefani G., Byrom M., Kelnar K., Ovcharenko D., Wilson M., Wang X., Shelton J., Shingara J. (2007). The let-7 microRNA represses cell proliferation pathways in human cells. Cancer Res..

[170] Yu F., Yao H., Zhu P., Zhang X., Pan Q., Gong C., Huang Y., Hu X., Su F., Lieberman J. (2007). let-7 Regulates Self Renewal and Tumorigenicity of Breast Cancer Cells. Cell.

[171] Grimm D., Wang L., Lee J.S., Schürmann N., Gu S., Börner K., Storm T.A., Kay M.A. (2010). Argonaute proteins are key determinants of RNAi efficacy, toxicity, and persistence in the adult mouse liver. J. Clin. Investig..

[172] Diederichs S., Jung S., Rothenberg S.M., Smolen G.A., Mlody B.G., Haber D.A. (2008). Coexpression of Argonaute-2 enhances RNA interference toward perfect match binding sites. Proc. Natl. Acad. Sci. USA.

[173] McBride J.L., Boudreau R.L., Harper S.Q., Staber P.D., Monteys A.M., Martins I., Gilmore B.L., Burstein H., Peluso R.W., Polisky B. (2008). Artificial miRNAs mitigate shRNA-mediated toxicity in the brain: Implications for the therapeutic development of RNAi. Proc. Natl. Acad. Sci. USA.

[174] Boudreau R.L., Martins I., Davidson B.L. (2009). Artificial MicroRNAs as siRNA shuttles: Improved safety as compared to shRNAs in vitro and In vivo. Mol. Ther..

[175] Beer S., Bellovin D.I., Lee J.S., Komatsubara K., Wang L.S., Koh H., Börner K., Storm T.A., Davis C.R., Kay M.A. (2010). Low-level shRNA cytotoxicity can contribute to MYC-induced hepatocellular carcinoma in adult mice. Mol. Ther..

[176] Singh A., Bhattacharyya N., Srivastava A., Pruett N., Ripley R.T., Schrump D.S., Hoang C.D. (2019). MicroRNA-215-5p Treatment Suppresses Mesothelioma Progression via the MDM2-p53-Signaling Axis. Mol. Ther..

[177] Hede K. (2010). MicroRNAs As Onco-miRs, Drivers of Cancer. J. Am. Med. Assoc..

[178] Ebert M.S., Neilson J.R., Sharp P. (2013). A MicroRNA sponges: Competitive inhibitors of small RNAs in mammalian cells. Nat. Methods.

[179] Carè A., Catalucci D., Felicetti F., Bonci D., Addario A., Gallo P., Bang M.L., Segnalini P., Gu Y., Dalton N.D. (2007). MicroRNA-133 controls cardiac hypertrophy. Nat. Med..

[180] Lindow M., Kauppinen S. (2012). Discovering the first microrna-targeted drug. J. Cell Biol..

[181] Umbach J.L., Cullen B.R. (2009). The role of RNAi and microRNAs in animal virus replication and antiviral immunity. Genes Dev..

[182] Gebert L.F.R., Rebhan M.A.E., Crivelli S.E.M., Denzler R., Stoffel M., Hall J. (2014). Miravirsen (SPC3649) can inhibit the biogenesis of miR-122. Nucleic Acids Res..

[183] Jopling C. (2012). Liver-specific microRNA-122. RNA Biol..

[184] Jopling C.L., Schütz S., Sarnow P. (2008). Position-Dependent Function for a Tandem MicroRNA miR-122-Binding Site Located in the Hepatitis C Virus RNA Genome. Cell Host Microbe.

[185] Baek J., Kang S., Min H. (2014). MicroRNA-targeting therapeutics for hepatitis C. Arch. Pharm. Res..

[186] Seto A.G., Beatty X., Lynch J.M., Hermreck M., Tetzlaff M., Duvic M., Jackson A.L. (2018). Cobomarsen, an oligonucleotide inhibitor of miR-155, co-ordinately regulates multiple survival pathways to reduce cellular proliferation and survival in cutaneous T-cell lymphoma. Br. J. Haematol..

[187] Gallant-Behm C.L., Piper J., Lynch J.M., Seto A.G., Hong S.J., Mustoe T.A., Maari C., Pestano L.A., Dalby C.M., Jackson A.L. (2019). A MicroRNA-29 Mimic (Remlarsen) Represses Extracellular Matrix Expression and Fibroplasia in the Skin. J. Investig. Dermatol..

[188] Reid G., Pel M.E., Kirschner M.B., Cheng Y.Y., Mugridge N., Weiss J., Williams M., Wright C., Edelman J.J.B., Vallely M.P. (2013). Restoring expression of miR-16: A novel approach to therapy for malignant pleural mesothelioma. Ann. Oncol..

[189] van Zandwijk N., Pavlakis N., Kao S.C., Linton A., Boyer M.J., Clarke S., Huynh Y., Chrzanowska A., Fulham M.J., Bailey D.L. (2017). Safety and activity of microRNA-loaded minicells in patients with recurrent malignant pleural mesothelioma: A first-in-man, phase 1, open-label, dose-escalation study. Lancet Oncol..

[190] Bouchie A. (2013). First microRNA mimic enters clinic. Nat. Biotechnol..

[191] Adams B.D., Parsons C., Slack F.J. (2016). The Tumor-Suppressive and Potential Therapeutic Functions of miR-34a in Epithelial Carcinomas. Expert Opin Ther Targets.

[192] Misso G., Di Martino M.T., De Rosa G., Farooqi A.A., Lombardi A., Campani V., Zarone M.R., Gullà A., Tagliaferri P., Tassone P. (2014). Mir-34: A new weapon against cancer?. Mol. Ther. Nucleic Acids.

[193] Ling H., Girnita L., Buda O., Calin G.A. (2017). Non-coding RNAs: The cancer genome dark matter that matters!. Clin. Chem. Lab. Med..

[194] Sergeeva O.V., Koteliansky V.E., Zatsepin T.S. (2016). mRNA Based Therapeutics—Advances and Perspectives. Biochemistry.

[195] Heiser A., Coleman D., Dannull J., Yancey D., Maurice M.A., Lallas C.D., Dahm P., Niedzwiecki D., Gilboa E., Vieweg J. (2002). Autologous dendritic cells transfected with prostate-specific antigen RNA stimulate CTL responses against metastatic prostate tumors. J. Clin. Invest..

[196] le Sage C., Lawo S., Cross B.C.S. (2019). CRISPR: A Screener’s Guide. SLAS Discov. Adv. life Sci..

[197] Jansen R., Van Embden J.D.A., Gaastra W., Schouls L.M. (2002). Identification of genes that are associated with DNA repeats in prokaryotes. Mol. Microbiol..

[198] Kurata M., Yamamoto K., Moriarity B.S., Kitagawa M., Largaespada D.A. (2018). CRISPR/Cas9 library screening for drug target discovery. J. Hum. Genet..

[199] Herrera-Carrillo E., Gao Z., Berkhout B. (2019). CRISPR therapy towards an HIV cure. Brief. Funct. Genomics.

[200] Beierlein J.M., McNamee L.M., Ledley F.D. (2017). As Technologies for Nucleotide Therapeutics Mature, Products Emerge. Mol. Ther. Nucleic Acids.

[201] Iwama H., Murao K., Imachi H., Ishida T. (2011). MicroRNA networks alter to conform to transcription factor networks adding redundancy and reducing the repertoire of target genes for coordinated regulation. Mol. Biol. Evol..

[202] Neilsen C.T., Goodall G.J., Bracken C.P. (2012). IsomiRs—The overlooked repertoire in the dynamic microRNAome. Trends Genet..

[203] Fehlmann T., Backes C., Kahraman M., Haas J., Ludwig N., Posch A.E., Würstle M.L., Hübenthal M., Franke A., Meder B. (2017). Web-based NGS data analysis using miRMaster: a large-scale meta-analysis of human miRNAs. Nucleic Acids Res..

[204] Lan C., Peng H., Mcgowan E.M., Hutvagner G., Li J. (2018). An isomiR expression panel based novel breast cancer classification approach using improved mutual information. BMC Med. Genomics.

[205] Telonis A.G., Magee R., Loher P., Chervoneva I., Londin E., Rigoutsos I. (2017). Knowledge about the presence or absence of miRNA isoforms (isomiRs ) can successfully discriminate amongst 32 TCGA cancer types. Nuc. Acids. Res..

[206] Hu W.Z., Tan C.L., He Y.J., Zhang G.Q., Xu Y., Tang J.H. (2018). Functional miRNAs in breast cancer drug resistance. Onco. Targets. Ther..

[207] Bayraktar R., Van Roosbroeck K. (2018). miR-155 in cancer drug resistance and as target for miRNA-based therapeutics. Cancer Metastasis Rev..

[208] Li M.J., Kim J., Li S., Zaia J., Yee J.K., Anderson J., Akkina R., Rossi J.J. (2005). Long-term inhibition of HIV-1 infection in primary hematopoietic cells by lentiviral vector delivery of a triple combination of anti-HIV shRNA, anti-CCR5 ribozyme, and a nucleolar-localizing TAR decoy. Mol. Ther..

[209] Digiusto D.L., Krishnan A., Li L., Li H., Li S., Rao A., Yam P., Stinson S., Kalos M., Alvarnas J. (2011). RNA-based gene therapy for HIV with lentiviral vector-modified CD34(+) cells in patients undergoing transplantation for AIDS- related lymphoma. Sci. Transl Med..

[210] Unwalla H.J., Li H.-T., Bahner I., Li M.-J., Kohn D., Rossi J.J. (2006). Novel Pol II Fusion Promoter Directs Human Immunodeficiency Virus Type 1-Inducible Coexpression of a Short Hairpin RNA and Protein. J. Virol..

[211] Canadian Agency for Drugs and Technologies in Health Pharmacoeconomic Review Report for Nusinsersin; Appendix 1, Cost Comparison. Nusinersen (Spinraza): (Biogen Canada Inc.): Indication: Treatment of patients with 5q SMA. CADTH Common Drug Review. https://www.ncbi.nlm.nih.gov/pubmed/30480926.

[212] King N.M.P., Bishop C.E. (2017). New treatments for serious conditions: Ethical implications. Gene Ther..

